# Applying deep learning on social media to investigate cultural ecosystem services in protected areas worldwide

**DOI:** 10.1038/s41598-024-64115-3

**Published:** 2024-06-13

**Authors:** Timothy Bing Lun Yee, L. Roman Carrasco

**Affiliations:** Department of Biological Sciences, National University of Singapore, 14 Science Drive 4, Singapore, 117543 Republic of Singapore

**Keywords:** Environmental impact, Machine learning

## Abstract

Protected areas (PAs) are the cornerstone of conservation efforts. Although they provide many benefits to humanity, the variability in the provision of cultural ecosystem services (CES) among global PAs remains unknown. To investigate this, we combined Convolutional Neural Networks with hierarchical clustering to categorize photos from Flickr taken in PAs worldwide. A final sample of 87,090 photos in 2813 PAs within 207 countries was obtained. Distinct global patterns of CES activities emerged. Such activities had three main interaction types: human-nature (abiotic), human-nature (biotic) and human–human. Human-nature (abiotic) interactions dominated in mountain ranges. Human-nature (biotic) photos were more common in equatorial countries, and human–human photos occurred mainly in Europe. To determine the extent of the influence of biome type of PAs on CES, mixed-effects models were subsequently run. These models additionally included the country of PAs as a random effect. Despite differences in physical environments, PAs within each country generally shared similar CES types. Moreover, the effect of biome differences was marginal, thereby demonstrating that country-level management of PAs likely has a more important role in influencing CES activities in PAs. To conclude, we suggest that our results demonstrate the utility of social media data for understanding visitor activities in PAs.

## Introduction

Protected areas (PAs) serve as important bulwarks to conserve biodiversity^1^. To ensure that PAs can achieve their function of maintaining species diversity, there is a need to devote sufficient funding and human resources^2^. Although many PAs traditionally rely on government funding for the maintenance of their ecosystems, the rising costs of their upkeep means that solely relying on such funding is becoming increasingly untenable^3^. As such, to provide an additional source of funds for effective maintenance of these PAs, many countries have progressively turned to encouraging nature-based tourism within them^4^. There are several ways that countries can collect such revenue from visitors. Although not commonplace, one such example is in the United States, with the National Park Service charging visitors an entry fee to certain PAs to fund conservations projects such as habitat restoration within those PAs^5^. With there being over 8 billion visits to PAs each year, there is over $600 billion in annual revenue from tourists^6^.

Nonetheless, increased tourism in PAs is also a double-edged sword. With such substantial numbers of tourists, it becomes especially crucial to monitor their movements to minimize their ecological impacts on particularly vulnerable species^7^. Understandably, tourism, in excess, has a detrimental effect instead, as overtourism having been found to unduly impact both flora and fauna present in PAs. There is thus a need to strike a balance between encouraging some tourism for increased revenue while also limiting its impact on the regions in PAs that are more sensitive to human activity^8^. Understanding the dominant activities present in a PA would hence enable more precise management of PAs to ensure that tourism remains sustainable^9^. Despite the burgeoning nature-based tourism industry, however, precise descriptions of specific activities undertaken in PAs worldwide remain largely unclear. Although there have been several studies^10–12^ that look at the interactions of humans with nature in PAs, they had limited spatial coverage as they only analyzed certain countries or regions, rather than highlighting global patterns. With PAs exhibiting great diversity in their physical environments, it is important to account for such variations.

To describe activities conducted in PAs in greater detail, it is first important to identify the Cultural Ecosystem Services (CES) that PAs can provide. CES provide “non-material benefits” to humans, such as “aesthetic, spiritual and psychological”^13^. Unlike other ES, the significance of CES to people cannot be understated, as many perceive them to be an important part of their own cultural identity^14,15^, and for improving their own physical and mental well-being^16^. However, despite their importance for humans, CES have generally been less understood in comparison to other ES types. Unlike other ES, the intangible and subjective nature of CES results in them being difficult to quantify^17^. As a result, current ES classification frameworks are perceived as being insufficient for encapsulating key features of CES.

With its widespread availability, social media can address existing gaps in CES identification from fieldwork^18^. Although fieldwork is more accurate due to its random sampling, it has limited spatial coverage due to workforce and budgeting constraints^19^. Social media thus provides a larger data pool to study human interactions with nature on an international level. As photos on social media are geotagged, they can serve as proxies to geolocate human activity within green and blue spaces^20^. Moreover, the presence of timestamps on photos allows the monitoring of changes in human activities at specific locations over time^21^. These two properties of social media photos consequently facilitate the conduct of retrospective studies over a long time period and wide spatial range^22,23^. Through such studies, we can then rapidly gather large amounts of human activity on a large scale^24^, thereby supplementing conventional data collection methods to unpack trends at the country level^25^.

As a result, social media enables us to describe patterns with sufficient resolution within countries, while also aggregating them at higher levels to observe larger regional or continental trends^12^. These studies then allow us to observe changes in various nature-related values over both time and space, such as life satisfaction^22^, subjective well-being^26^ and types of activities in nature^22,27^.

Despite the widespread availability of social media, most of these analyses tend to be bounded within certain countries or regions. For instance, the comprehensive overview of CES types determined by mining a large sample of photos by Egarter Vigl et al.^11^ only studied one PA, The Dolomites, in Italy. Also, the study by Richards and Tunçer^27^ uncovered some insightful findings of the CES types of activities undertaken by people but limited their coverage to Singapore. It is thus worthwhile to determine whether these analyses can be easily scaled up, and whether such trends will still hold at larger spatial ranges.

Nevertheless, social media mining alone is insufficient to overcome bottlenecks in data analysis. To make sense of the large amount of data collected, they have to first be processed. To overcome this mismatch between the speed of data collection and analysis, machine learning can be used in tandem with social media. To meet the needs of automated object recognition, one particular type of machine learning models is relevant: Convolutional Neural Networks (CNNs). Some examples of commercially available CNNs that were used to classify photos in nature include Google's Cloud Vision API^22,27^ and Microsoft's Azure Cognitive Services^28^. As compared to manual labeling, these models remained highly accurate in identifying human activities in nature^22,27^.

Bearing in mind the benefits afforded by social media and machine learning for data collection and processing, we aim to tap on these tools to accelerate and scale-up current initiatives towards CES identification. Following which, we aim to understand the variability in CES across PAs as a function of ecological and institutional contexts. Here we answer the following research questions: How similar are PAs around the world in their provision of CES types? How far can variations in CES across PAs be explained by biome type and country?

We do this through four main stages: data collection and cleaning, pre-processing of photos, GIS analysis of CES, and statistical analysis of biome types. The initial data collection involved gathering a sample of photos taken in PAs from publicly available user accounts on Flickr. The photos were then processed before CES identification by using CNNs to first describe the main object of focus in each photo using content tags. Following which, hierarchical clustering was done to group photos with similar activities. After this, GIS analysis of CES was done by manually identifying the predominant CES type in each photo. Then, similarities and differences within, and across, countries in the global distribution of CES types across PAs were noted. Statistical analysis of biome types and country-level variation was finally performed.

## Methods

### Protected areas shapefile generation

The initial set of PAs was obtained from the World Database on Protected Areas dated May 2022^29^. The criteria for retaining PAs were those of designated (i.e., official) status, had an associated polygon, and were of terrestrial type. There were 246,483 PAs that met these criteria.

### Extracting photos from Flickr

The above subset of PAs was then used as bounding boxes in querying Flickr using its official API via the photosearcher package in R^30^. To get a sample of photos in each country, PAs per country were subset to only retain the largest 40 PAs for photo searching. Additional search criteria included photos geotagged with coordinates within the PAs and that were taken within the time period from 1 January 2010 to 31 December 2021. In addition, only photos in the Public Domain or that had a Creative Commons license were retained. This ensures that only photos that were made public by their users and whose users had given consent to be downloaded were downloaded. This searching and downloading of photos from Flickr was approved by the National University of Singapore Institutional Review Board via an expedited ethics review (approval number: NUS-IRB-2022-330). From these criteria, Flickr search found a total of 824,508 photos across 207 countries and administrative regions.

For computational reasons, a cut-off of 2000 photos for each country was imposed. Countries that had more than this number of photos had a sample of photos randomly sampled from all the photos in that country. The sampling method was done by random sampling within each PA in each country, and the number of photos to retain from each PA was the cut-off divided by the total number of sampled PAs within each country. In addition, PAs that still had more photos than the above cutoff had their photos further randomly sampled to limit one photo from each user. This ultimately produced a subset of 107,750 photos.

### Photo label annotation

The above subset of photos was then downloaded using the photosearcher package in R. As only the photos themselves were used in downstream analyses, their metadata and captions were removed. All photos were then processed via Microsoft’s Azure Computer Vision API for tagging. This was done to identify potential labels that could be later used to describe the most common activities in the sample of photos. The API was accessed via the AzureVision package in R^31^. Images submitted to the API return a list of content tags describing “the objects, living beings, and actions identified” within them, together with a confidence score for each tag^32^. A cut-off of retaining only tags which had more than or equals to 0.5 confidence was used. After excluding invalid photos and photos that failed to return any tags, 5127 tags were identified from 87,090 photos that were processed.

### Hierarchical classification into activities

The photos and their tags were then converted into a sparse matrix with each photo being one row and each tag being one column. The tag information for each photo was stored in a manner whereby each tag that was present in a photo had a “1” in its corresponding cell, while each tag that was absent in a photo had nothing in its corresponding cell. The sparse matrix was then converted to a distance matrix for hierarchical classification using the wordspace package in R^33^. The distance measure used was Jaccard distance.

Hierarchical agglomerative clustering was applied to the distance matrix using complete-linkage clustering through the fastcluster package in R^34^. Hierarchical clustering was used for cluster analysis as it is an established method favored by several studies^22,27^ to classify photos by CES types. The clustering process then generated a dendrogram which was consulted to determine the number of optimal clusters to cut. As there was difficulty in judging the optimum number of clusters from the dendrogram alone, it was then decided, as a first step, to cut the tree at its maximum height (h = 0.999), thereby generating 430 clusters.

Subsequently, further manual processing was undertaken to merge similar clusters. This was motivated by the observation of many clusters containing a high degree of overlap in their top 10 photo tags. The degree of similarity used to determine overlapping clusters was fixed to be at least 5 similar tags in both clusters. Clusters with less than 10 tags and clusters with low photo counts were then merged into an “OTHERS” cluster. Clusters whose photos did not describe a distinct object were also merged into this “OTHERS” cluster. After these steps, 102 clusters remained. Each of these remaining clusters was then labeled with the most common object described in its photos, as observed from its top 10 tags.

### Identification of CES types

The categories of the latest version of Common International Classification of Ecosystem Services (CICES, Version 5.1)^35^ were then consulted to identify possible CES categories to label image clusters. However, upon closer inspection, some of the definitions for CES categories in CICES were deemed to be inadequate in describing the observed activities in the social media photos. Consequently, we decided to propose our own CES categories by modifying the existing CES definitions in CICES. There are three categories proposed by us that are used within this paper: Biotic, with subcategories Transport, Sports and Watching; Abiotic with subcategories Watching; and Humans with subcategories Heritage and Socializing (Supplementary Tables S2–S4 provide the definitions for each category and a comparison with the CICES definition).

There are a few notable changes we made in our CES definitions as compared to their definitions in CICES. One such change is the addition of a third CES category: Humans. Although CICES mainly proposed only two types of CES (“Biotic” and “Abiotic”), we decided to also include an additional category (“Humans”). This was after finding an unexpectedly high number of photos depicting activities between humans (see Supplementary Fig. S1E,F for an example), rather than between humans and nature. As this category was absent in CICES, to more accurately capture such nuances, it was decided to expand the proposed CES categories in this paper beyond that of those present in CICES. Definitions of this category were adapted from those previously proposed by Chan et al.^14^. As argued in the study, aside from the intrinsic and instrumental values of nature, there is an additional category of values that nature can bring for humans: relational values. These values in particular aim to capture the relationships “between people but involve nature”, and contain two subcategories: “involving the human collective”, and “primarily individual”^14^. Such values in particular are of interest in describing the patterns of human interactions observed in the dataset.

Another change we made was moving the Sports category (Supplementary Table S2) from Biotic to Abiotic. Despite CICES suggesting that such sporting activities should fall under Biotic CES, we decided to present them under Abiotic CES instead. This is because such activities generally appear to not prioritize the direct interactions of humans with the biotic aspects of the environment, and instead focus more on the abiotic aspects.

### Correlates of CES categories

To determine whether there was any relationship between the biome type that a photo was taken in and its CES type, generalized mixed-effects models were fitted to model their relationship. All models were generated in lme4^36^. Each model was fitted at the individual photo level, and had the CES category of each photo as the outcome variable. As each CES category was coded as either being present (“1”) or absent (“0”) for each photo, a binomial family was used for all models. With there being three different CES categories (Abiotic, Biotic, and Humans), three separate models were constructed.

To determine the biome type of each photo, the IUCN Global Ecosystem Typology 2.0 was referenced^37^ using the sf package in R. For each model, the biome type of each photo at the smallest (functional) level was the fixed effect. Only the top 6 biomes were retained as levels in the biome type predictor, with all other biome types being classified as “OTHERS” (Supplementary Table S5).

The PA of each photo nested within its country were included as random intercepts. The inclusion of the random effect was to determine how the odds of a photo belonging to a certain CES varied at the country-level. To ensure models converged, the number of points for evaluating the Adaptive Gauss-Hermite Quadrature was set to 0 (nAGQ = 0), and the nloptwrap optimizer was used.

## Results

### CES cluster labels

**Figure 1:**
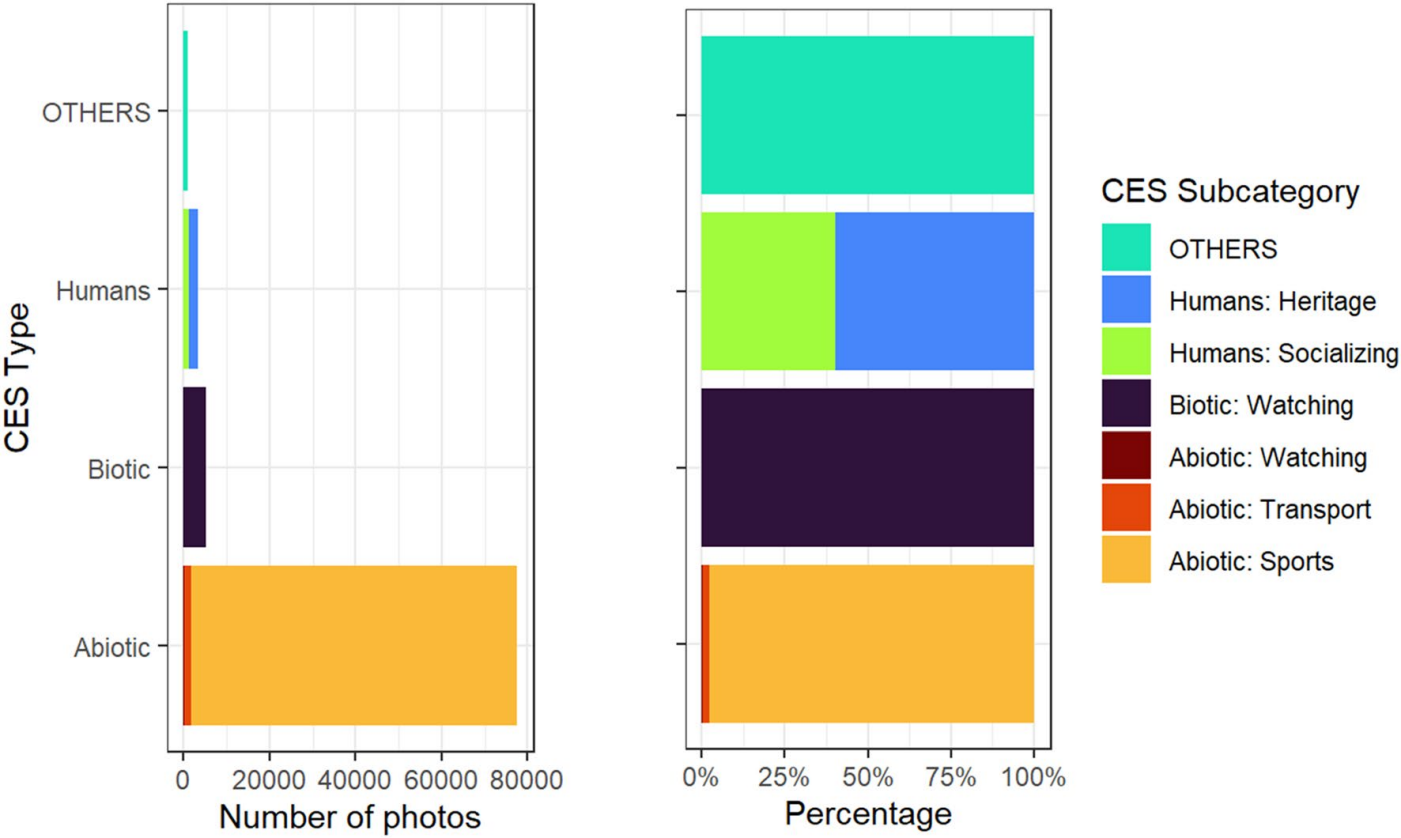
Number of photos (left) and percentage (right) for each CES category and subcategory in the final sample of photos.

The dominant CES type were Abiotic photos (89.1%), where most (97.6%) of these photos were in the Sports subcategory, followed by the Transport (2.11%) and Watching (0.33%) categories. The second largest CES type were Biotic photos (6.00%), and then Humans photos (3.74%). Of the Humans photos, most photos were in the Heritage subcategory (59.7%), and the remainder were in the Socializing subcategory (40.2%). A small number of photos (1.21%) had tags not clearly related to humans or nature categorized as “OTHERS” (Fig. 1).

As indicated by the top 10 tags of each CES subcategory (Supplementary Fig. S1), subcategories within the same CES category tended to resemble each other more than subcategories from other CES categories. Both Humans subcategories had tags that referenced human-made objects (e.g., “building” in Heritage and “furniture” in Socializing). Likewise, the three Abiotic subcategories were similar in their tags that described the physical environment (e.g., “road” in Transport and “astronomy” in Watching); this was unlike the Watching subcategory of Biotic CES, which was biotic in its animal and plant tags (e.g., “aquatic bird” and “animal”). Detailed definitions of the underlying descriptors used to categorize photos into Abiotic, Biotic, and Humans can be found in Supplementary Tables S2–S4.

### Geographic distribution of photos

**Figure 2:**
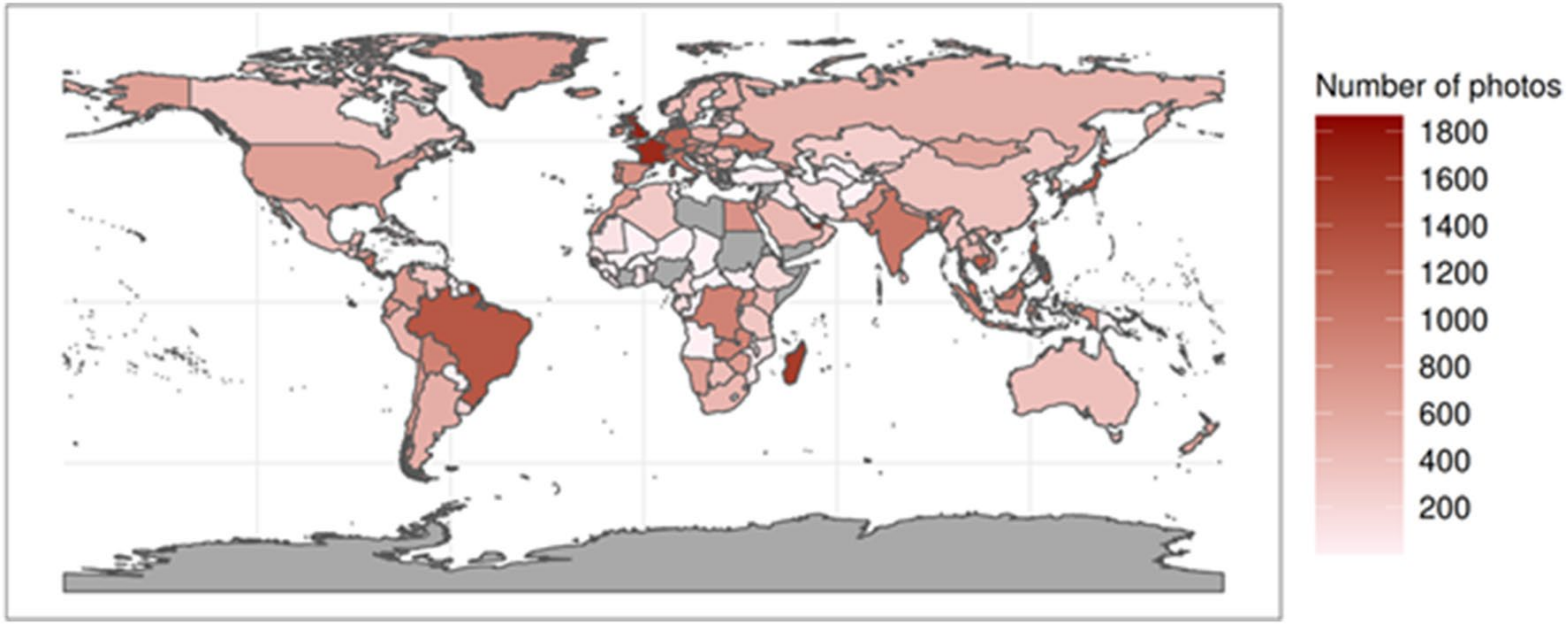
Number of photos in each country. Grey indicates absence of photos.

The final sample of photos was taken in 2813 PAs within 207 countries and administrative regions (Fig. 2, Supplementary Figs. S2, S3). Most photos worldwide were mainly Abiotic CES type, regardless of country. Such photos were especially dominant near temperate and polar regions. The main focus for visitors to these PAs appears to be their physical environments with an emphasis in activities within mountain ranges (e.g. PAs in Japan and New Zealand, Supplementary Figs. S4, S5 respectively).

Most photos with Biotic CES type were in countries near the equatorial belt, regardless of continent. These photos occurred in PAs that contained high species richness and endemism, such as those Indonesia (Supplementary Fig. S6) and in Ecuador, bordering the Amazon Rainforest (Supplementary Fig. S7). Conversely, most photos with Humans CES type were near Europe. These photos were related to national parks containing villages or human-made sites with important historical value (Supplementary Fig. S8).

There were distinct variations in CES type in PAs both within countries, and across neighboring countries. One example is in southern Africa. Botswana had a PA, Moremi Game Reserve (B), that contains photos of primarily Biotic CES type, while nearby South Africa has a PA, Kruger National Park (C), that almost solely had photos of only Abiotic CES type (Supplementary Fig. S9), showing high specialization in the type of CES offered by each PA.

### Correlates of CES categories

**Figure 3:**
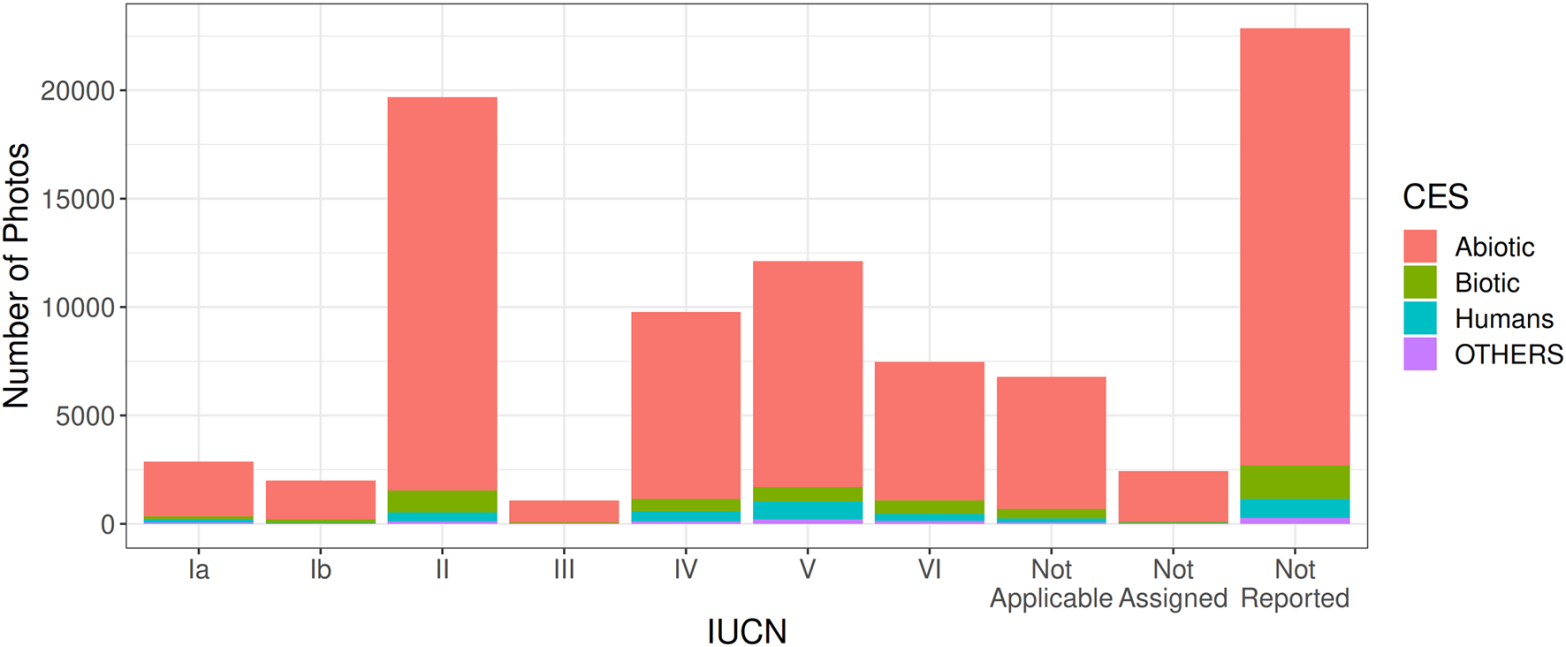
Number of photos in sampled PAs sorted by IUCN category, for all CES types.

When separating PAs by their IUCN classification, a clear trend emerges (Fig. 3). Most of the photos appear to be taken in PAs with IUCN Category II (National Park), with there being comparatively fewer photos taken in PAs with IUCN Category Ia (Strict Nature Reserve) and Category Ib (Wilderness Area). There also appears to be a significant number of photos taken in PAs with unclear (Not Reported or Not Assigned) designations by IUCN. Such trends continue to persist across all three CES types (Supplementary Figs. S10–S12).

There were 68 different functional groups of 21 different biomes present in photos. As defined in the IUCN Global Ecosystem Typology 2.0^37^, functional groups here refer to “a group of related ecosystems within a biome that share common ecological drivers, which in turn promote similar biotic traits that characterise the group”. Most photos were either in intensive land-use sites (T7) or palustrine wetlands (TF1) (Supplementary Fig. S13).

**Figure 4:**
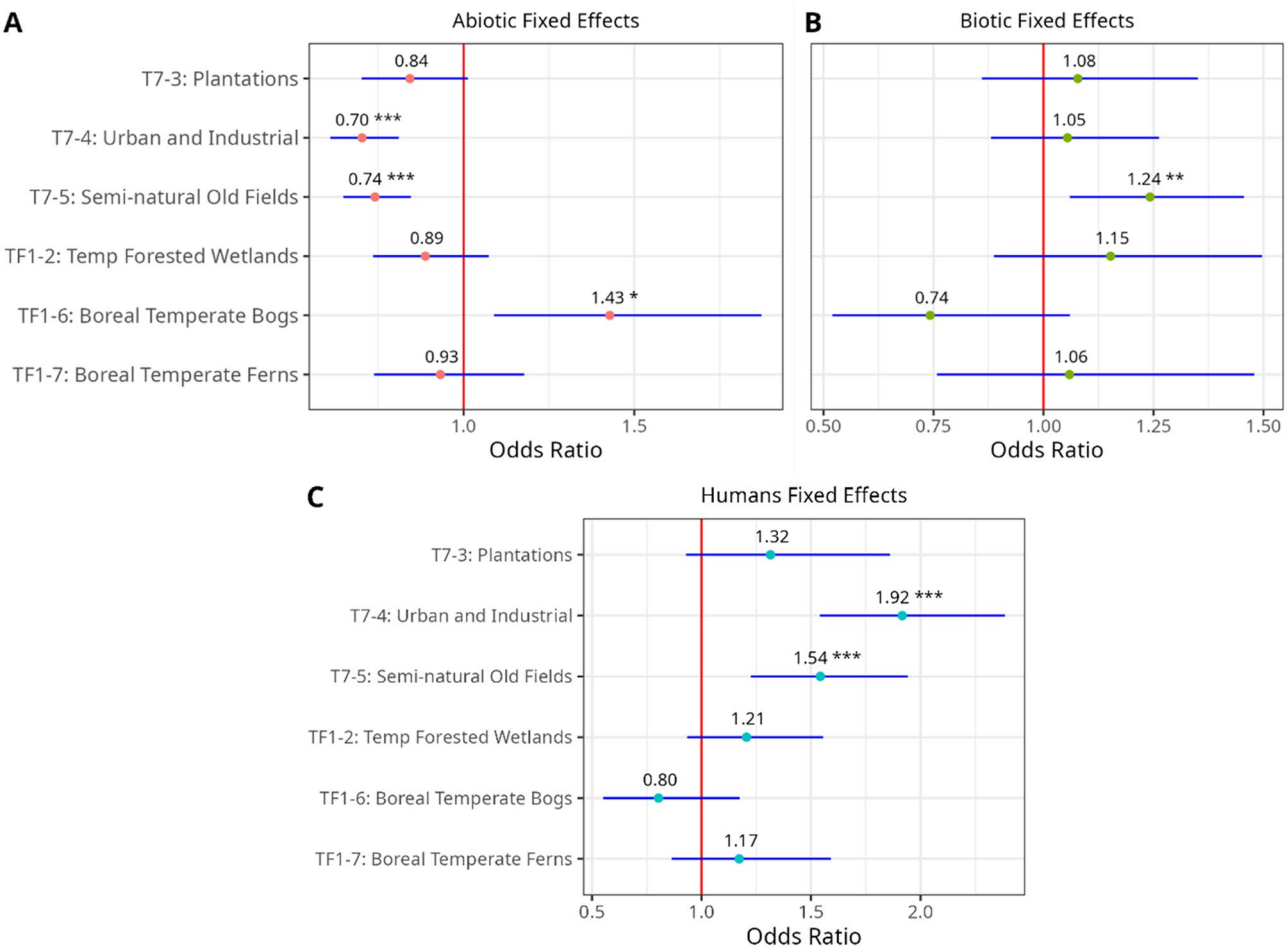
Fixed effects of biome type of generalized linear mixed-effects models for abiotic CES type (**A**), Biotic CES type (**B**), and humans CES type (**C**). Error bars (in blue) for all predictors indicate a 95% confidence interval. Only significant predictors are labelled: * 0.01 < *p* ≤ 0.05, ** 0.001 < *p* ≤ 0.01, *** *p* ≤ 0.001.

For Abiotic CES type (A), photos in boreal temperate bogs (TF1-6) were significantly more likely to be Abiotic CES type, while photos in urban and industrial (T7-4), and semi-natural old fields (T7-5), were significantly less likely to be Abiotic CES type. For Biotic CES type (B), photos in semi-natural old fields (T7-5) were significantly more likely to be Biotic CES type. For Humans CES type (C), photos in urban and industrial (T7-4), and semi-natural old fields (T7-5) were significantly more likely to be Humans CES type (Fig. 4).

The amount of variance explained by biome types on predicting each of the CES types was low. The conditional R^2^ was much larger than the marginal R^2^ for all three models (Supplementary Table S1). This indicates that country-level differences explained substantially more variability on CES types than biome type.

**Figure 5:**
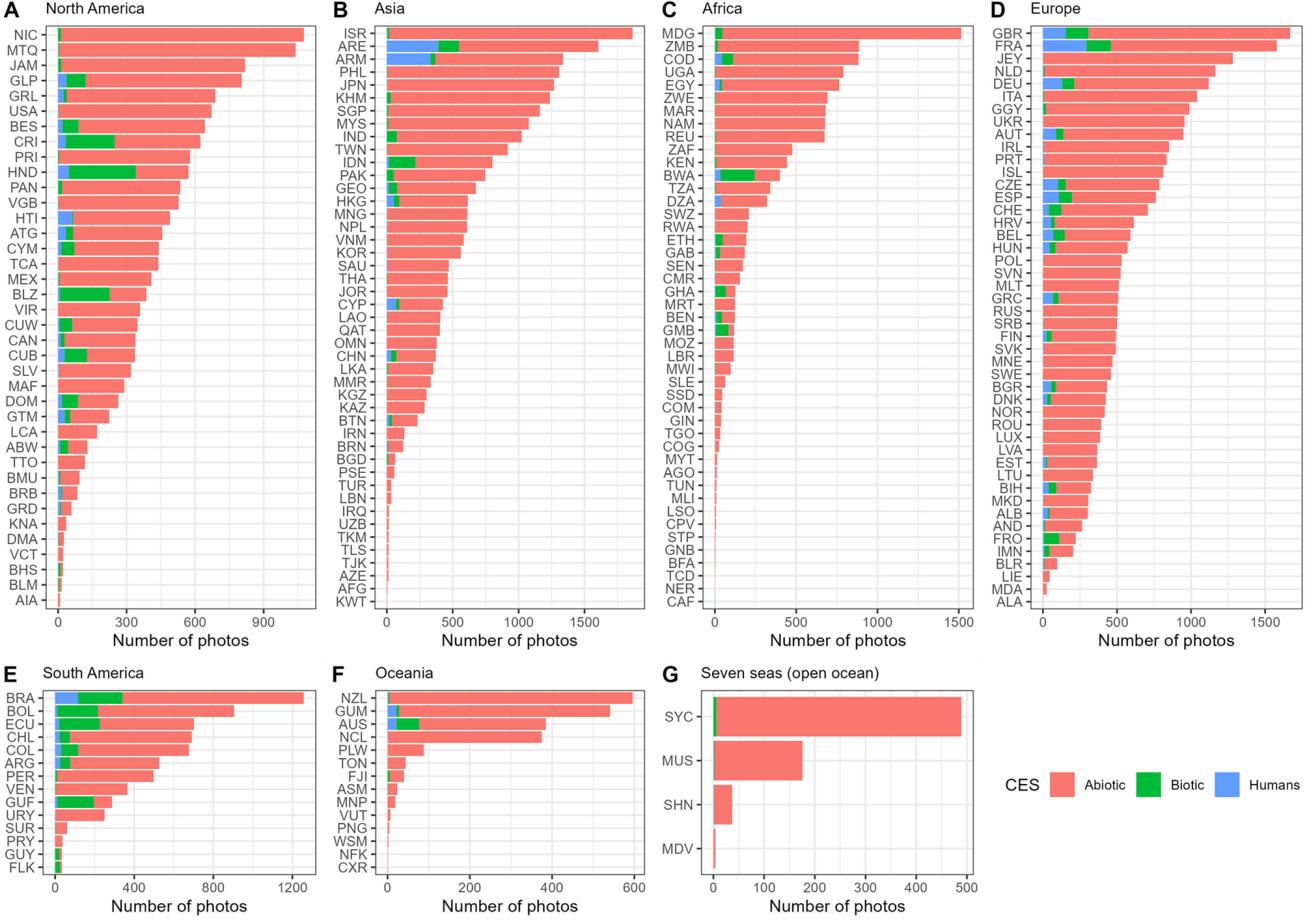
Number of photos of each CES category in each country separated by continent. Countries are referenced by their ISO 3166-1 alpha-3 codes. Detailed breakdown by individual CES types are in Supplementary Figs. S17–S21.

Countries with more positive random intercepts for Abiotic CES type (Supplementary Fig. S14) tended to have more negative random intercepts for both Biotic (Supplementary Fig. S15) and Humans (Supplementary Fig. S16) CES types, and vice-versa. This trend was present regardless of the continent. General trends in random intercepts for all CES types reflect the trends in their numbers across countries (Fig. 5). Countries with higher proportions of photos with Biotic CES types (e.g., Indonesia in Asia), tended to have positive and larger random intercepts in the Biotic mixed-effects model (Supplementary Fig. S15). Just as previously identified, these countries also tended to be near the equator.

## Discussion

The results provide a global overview of CES types in PAs, highlighting their sheer diversity across countries. Unexpectedly, a large proportion of photos in the dataset appeared to describe activities of PAs in mountainous regions. This supports previous findings that PAs are generally “high and far”, which means that they tend to be further from urban regions and roads due to their higher altitude^38^. However, contrary to the expectation that these “high and far” PAs are inaccessible, we identified such PAs to instead be rich in their provision of CES types, as noted by the many photos of people engaging in alpine sports.

We also observe other distinct trends in CES types among spatially related PAs. There is a greater proportion of photos with Biotic CES type in equatorial countries, with the opposite trend being observed at temperate countries. These trends align well with expected traits of physical environments in PAs. For instance, the many sites of important historical value being located within PAs in Europe could help explain the finding of a larger proportion of photos with Humans CES type being detected. In addition, the location of most of the biodiversity hotspots in the world being in equatorial countries^39^ correlates well with the finding of higher proportions of photos with Biotic CES type in these regions.

Although PAs vary widely in their physical environments, we find that these differences may have a smaller than expected influence on the dominant CES type in each PA. Instead, PAs within each country tend to have a high degree of overlap in CES types, even if they differ in biome types. Such observations were found from the mixed-effects models using biome type as fixed effects and country of PAs as random effects to predict the CES types in PAs. As seen in Supplementary Table S1, the conditional R^2^ was much larger than the marginal R^2^ for all three CES types, thereby indicating that country-level differences are more important than the biome type of PAs in explaining variation in CES types across PAs. Such findings resemble a previous study that aimed to identify CES activities from social media data among countries in Europe by van Zanten et al.^40^. In it, they found that country differences were able to explain more variance in CES as compared to geographical predictors, such as accessibility and terrain.

Such observations could be explained by the process with which PAs get designated. Different land use policies for all PAs within each political boundaries for ease of administration occur. Subsequently, this then results in country-specific land use management guidelines having a strong influence on the provision of available CES types in PAs^41,42^. Such differing guidelines across countries can then cause PAs in different countries that share similar biome types to diverge in their CES types. For instance, due to Norway having a more liberal conservation policy than Canada, this meant that a wider range of human activities (such as camping and fishing) were allowed in Norway, as opposed to in Canada^43^. As such, it is important to also consider the potential contribution of its country’s land use management guidelines when attempting to explain the existence of certain CES in a PA. Another potential explanation is the different preferences and values towards the use of PAs held across countries. For instance, in a public ecosystem services mapping survey, Norwegians mapped more values related to hunting/fishing and gathering than Polish respondents focusing on scenery, biodiversity and water quality in the ecosystem services they attribute to protected places^42^. Such differences in CES can also vary depending on the cultural values ascribed to nature by different countries. For instance, although Chinese visitors appear to share many similarities with Western visitors in the CES they attribute to protected places, they appear to differ in their expectations with regards to wildlife interactions and birdwatching^44^. Consequently, it is thus pertinent to additionally consider the cultural component of CES and how they differ across countries.

Additionally, another notable observation is that most photos taken in PAs tend to be from IUCN Category II (National Park), with the number of photos in PAs of IUCN Categories Ia (Strict Nature Reserve) and Ib (Wilderness Area) being comparatively fewer (Fig. 3). As expected, the PA categories which have stricter protections against human intervention also face less footfall from visitors due to tighter restrictions on their entry. However, there is still a notable number of photos found in these stricter IUCN PA categories, thereby indicating that the human footprint in these areas may be higher than expected. Similar findings were observed by other studies of global PAs^45,46^. Nonetheless, Leberger et al.^47^ noted that the stricter IUCN categories still experienced lower forest loss than other categories, despite the higher than expected human pressure exerted on them. Beyond this observation, another finding from our dataset is that there is a significantly large number of photos from PAs with unclear IUCN designation, although not having an IUCN classification, these PAs have been designated as official PAs by their respective countries’ governments and demonstrate an important role in the provision of cultural ES. As cautioned by others^48,49^, some of the IUCN categories, especially V (Protected Landscape or Seascape) and VI (Protected Area with Sustainable Use of Natural Resources), tend to be ambiguous in their definitions, and also tend to be less strictly monitored on their condition. Such constraints subsequently limit our capacity to elucidate the patters of activities performed in these categories.

More prominently, in this study, the major CES activities observed in PAs tended to align with the intended activities that the countries are using to attract visitors to those PAs. For instance, both the Komodo National Park in Indonesia and the Moremi Game Reserve in Botswana have high proportions of photos of Biotic CES type taken in them. This supports previous findings where activities identified on social media were found to agree strongly with the actual activity preferences of visitors to PA as previously surveyed^50,51^. For example, Hausmann^50^ noted that both the surveys and social media data collected within Kruger National Park in South Africa identified that the most favoured subject in photos taken by visitors are the charismatic, large-bodied mammals present within the national park.

Consequently, such high levels of concordance observed between movement patterns of individuals on social media and physical surveys^52^ underscores the possibility of social media as an alternative tool for local governments for monitoring trends in human activities in PAs. By tracking dominant CES in PAs, countries can hence uncover the precise activities undertaken in those areas. Among the different activities that could be undertaken in PAs, there are some which are more damaging to the environment, such as mountain biking instead of hiking^53^. As such, if there are any notable changes in CES types towards those with greater environmental impact, constant monitoring of social media posts would enable countries to react more rapidly to such marked increases in land use degradation by enacting measures such as targeted temporary closure of those areas. Social media mining thus has utility for guiding country-level land-use management to ensure that PAs remain conserved.

There were three main categories of CES observed in this study: Biotic, Abiotic, and Humans. Unlike the CES categories proposed by CICES (Supplementary Tables S2 and S3), the addition of Humans CES type in the proposed definitions in this paper appears to have been previously overlooked. To incorporate this proposed third dimension of CES, we suggest that CES definitions could be reframed by considering the relational values that people receive from their interactions with nature^14^. Such a modification to Humans CES categories is also supported by studies of CES in PAs. Egarter Vigl et al.^11^, using a survey of photos by visitors to The Dolomites in Italy, identified four main categories of CES: “aesthetic value, outdoor recreation, cultural heritage, symbolic species”. The “outdoor recreation” and “cultural heritage” are closely linked to Humans CES instead. Similar findings were also present in Cardoso et al.^10^, where an analysis of social media posts revealed that users value “aesthetics appreciation” in Peneda-Gerês in Portugal, while they value “cultural heritage and spiritual enrichment” in Sierra Nevada in Spain.

Another finding is that CES definitions made from objective descriptions of visible objects in photos are more relevant for CES identification from social media. Such an incongruity between proposed and observable activities was also present in a similar study by Richards and Tunçer^27^. They found that photos could only be classified according to their dominant object, which fell into the categories of “Transport, Plants, Animals, Food, People, Sports, Landscape and miscellaneous”. This limited applicability of CES definitions was also recognized as one limitation of the current CICES framework^35^. However, making CES definitions more concrete potentially deemphasizes the emotional and cognitive evaluations that attract people to nature and misses the underlying motivations for humans to take photos in nature; there is thus a danger of losing important information about CES types^27^. To circumvent this subjectivity in photo interpretation, other forms of information could also be concurrently collected. By using a hybrid method of combining both photo metadata and text data, estimation of the intent of people who visit a protected place may be more accurate^54^.

Our study had several limitations. Firstly, the most notable limitation is that the entire dataset was sourced from Flickr alone, with no corresponding fieldwork studies done to corroborate any findings. This consequently has implications on the ability of the users in the sampled dataset to be a robust representation of their respective country’s populations. In general, users of Flickr tend to be skewed towards a certain kind of demographic, that being younger than the average visitor age to PAs^27^. Additionally, as the photos of animals uploaded onto social media platforms tend to be taken by specialized equipment, there is also a gap in availability of the necessary digital devices required to obtain good quality photos, with such infrastructure being less available in developing countries^55^. As such, there is need to correct for such biases in the data collected before generalizing any findings from social media towards actual visitor habits in PAs. One such possible method could be to consider a twin approach of combining both big data analytical methods with conventional fieldwork approaches. Such fieldwork can involve conducting interviews with people visiting the PAs to validate the CES activities identified from social media^56^.

Secondly, in our Flickr dataset, although there are a high number of photos available for developed, English speaking, countries, the number of photos in less developed and/or non-English speaking countries is much fewer. Most prominently, there are a number of countries with low number of photos counts (Supplementary Figs. S14, S16 and S17). For instance, there were only 85 photos found in PAs located within Barbados, in comparison to the countries with the highest (Nicaragua, 1076 photos) and lowest (Anguilla, 9 photos) photo counts in North America (*M* = 380, *SD* = 286). This glaring disparity in available photos across countries on Flickr highlights the shortcoming of using social media for sourcing photos: there is unequal international coverage. As highlighted in Supplementary Fig. S3, there appear to be distinct clusters of countries with significantly more photos than other regions in the world. For instance, European countries (*M* = 591, *SD* = 376) such as France have disproportionately more photos (1579 photos) than African countries (*M* = 262, *SD* = 336) such as the Gambia (118 photos). Additionally, as the background profile of the users may not be clear at times, it is uncertain as to whether most visitors are indeed representative of the local population, or that they tend to be overseas visitors making short trips abroad; this is particularly pertinent when considering the photos on social media taken in developing countries^57^. To better capture the activities of the local population, future studies may hence wish to consider using more than one social media platform, with a particular emphasis on regional social media sites. For instance, the recent study by Wang et al.^58^ mined social media posts on Weibo in China to uncover CES in Xiamen, which would give a more accurate insight into the activities of locals.

Lastly, another limitation of this study is that we chose the number of clusters and matched them with the CICES classification manually. Despite this approach, most of the clusters successfully captured common activities that could describe the labeled objects in each cluster. In addition, the resolution of the CES types identified in PAs through machine learning and social media is sufficiently high in identifying dominant CES types between neighboring PAs. These findings are supported by past studies^9^. As such, with social media and machine learning having utility in sampling from a wider spatial range without losing much accuracy^18^, this combination is viable for addressing existing gaps in manually collected data.

In conclusion, we observed clear trends in CES types in PAs across countries and continents. While the Abiotic CES type dominates most PAs worldwide, there are distinct clusters for other CES types, such as equatorial countries for the Biotic CES types, and European countries for the Humans CES type. This study demonstrates that pairing social media with machine learning is a viable approach for large scale socio-ecological data analyses. Additionally, despite their diversity of physical environments, PAs within each country were found to have similar CES types. These CES types were also found to match similar nature-based recreational activities as marketed by these countries. Ultimately, with such concordance in CES types having been uncovered, this study has highlighted the usefulness of social media for monitoring global distributions of land use patterns in PAs.

### Supplementary Information

Supplementary Information.

## Data Availability

The data that support the findings of this study are openly available as a repository deposited at figshare at https://figshare.com/articles/dataset/Global_CES_in_PAs/24047613.
