# Supplementary material for: Applying deep learning on social media to investigate cultural ecosystem services in protected areas worldwide
Source: Sci Rep. 2024 Jun 13;14:13700. doi: 10.1038/s41598-024-64115-3 (PMC11176381; doi:10.1038/s41598-024-64115-3)
Supplement: Supplementary file 1 — Supplementary Information. [file 41598_2024_64115_MOESM1_ESM.pdf]

**Applying deep learning on social media to investigate cultural ecosystem services in protected areas worldwide**

Timothy B.L. Yee[1,*], L. Roman Carrasco[1]

[1]Department of Biological Sciences, National University of Singapore, 14 Science Drive 4, Singapore 117543, Republic of Singapore.

[*]Email: timothyybl@u.nus.edu. Tel:. Fax: +65 67792486.

**A** Abiotic (Transport)

| Top 10 AI-generated tags (decreasing order) | | | | |
|---|---|---|---|---|
| **1** vehicle | **3** outdoor | **5** car | **7** road | **9** auto part |
| **2** land vehicle | **4** wheel | **6** sky | **8** tree | **10** tire |

| Manually Identified Objects | | |
|---|---|---|
| Driving | Train | Train station |
| Flying | Hot air balloon | Car |

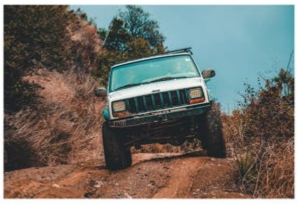

**B** Abiotic (Sports)

| Top 10 AI-generated tags (decreasing order) | | | | |
|---|---|---|---|---|
| **1** outdoor | **3** nature | **5** mountain | **7** landscape | **9** plant |
| **2** sky | **4** tree | **6** water | **8** cloud | **10** grass |

| Manually Identified Objects | | |
|---|---|---|
| Mountain Climbing | Watersports | Cycling |
| Hiking | Skiing | Cave |
| Scuba Diving | | |

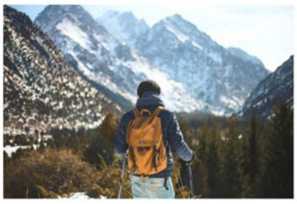

**C** Abiotic (Watching)

| Top 10 AI-generated tags (decreasing order) | | | | |
|---|---|---|---|---|
| **1** astronomy | **3** moon | **5** star | **7** outdoor | **9** sun hat |
| **2** sky | **4** night | **6** hat | **8** fashion accessory | **10** clothing |

| Manually Identified Objects | |
|---|---|
| Stargazing | Lake |
| Walking Outdoors | Mountain Fog |

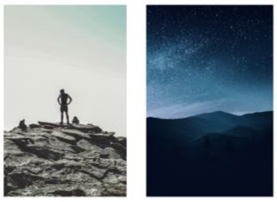

**D** Biotic (Watching)

| Top 10 AI-generated tags (decreasing order) | | | | |
|---|---|---|---|---|
| **1** animal | **3** bird | **5** branch | **7** wildlife | **9** perched |
| **2** outdoor | **4** tree | **6** water | **8** plant | **10** aquatic bird |

| Manually Identified Object | | | | |
|---|---|---|---|---|
| Bird | Butterfly | Reptile | Palm Tree | Mushroom |
| Gardening | Monkey | Wildlife | Fruit Tree | Animal |

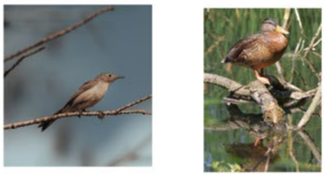

**E** Humans (Heritage)

| Top 10 AI-generated tags (decreasing order) | | | | |
|---|---|---|---|---|
| **1** building | **3** sky | **5** window | **7** cloud | **9** old |
| **2** outdoor | **4** house | **6** tree | **8** stone | **10** church |

| Manually Identified Object | | |
|---|---|---|
| Old Building | Old Bridge | Urban Exploration |
| Old Statue | Walking Urban | Old Ruins |
| Old Church | Art | Museum |

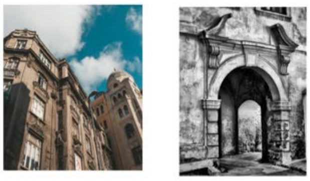

**F** Humans (Socializing)

| Top 10 AI-generated tags (decreasing order) | | | | |
|---|---|---|---|---|
| **1** indoor | **3** drawing | **5** table | **7** chair | **9** text |
| **2** food | **4** painting | **6** art | **8** floor | **10** furniture |

| Manually Identified Object | | | | |
|---|---|---|---|---|
| House | Cooking | Beach | Market | Dining |
| Art | Beer Drinking | Camping | Belly Dancing | Birthday Celebration |

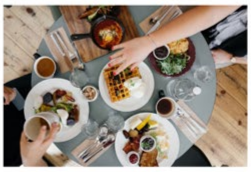

**Supplementary Figure S1. The top 10 tags as identified by AI, arranged in decreasing order (top), all identified objects that were manually interpreted (middle), and example photo(s) (bottom), for each CES subcategory (A to F).** Example photos were from pexels.com, a free stock photo website where photos can be used without attribution. Due to privacy concerns, no photos from the actual Flickr sample are shown.

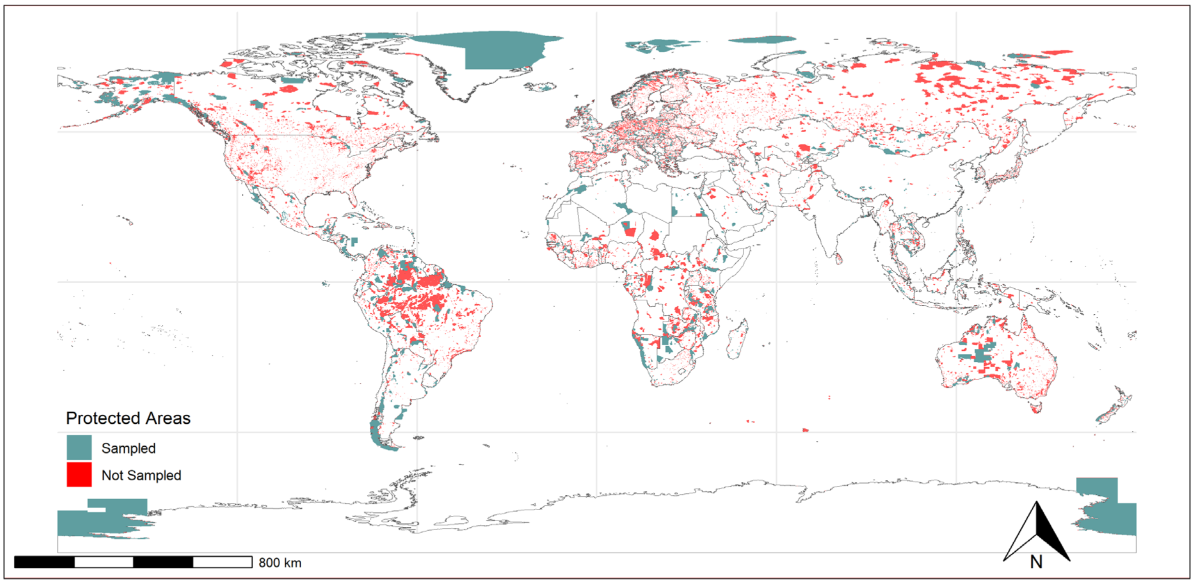


**Supplementary Figure S2. Global map of all PAs that were in the WDPA (May 2022) with those that were sampled (in green), and those that were not sampled (in red).**

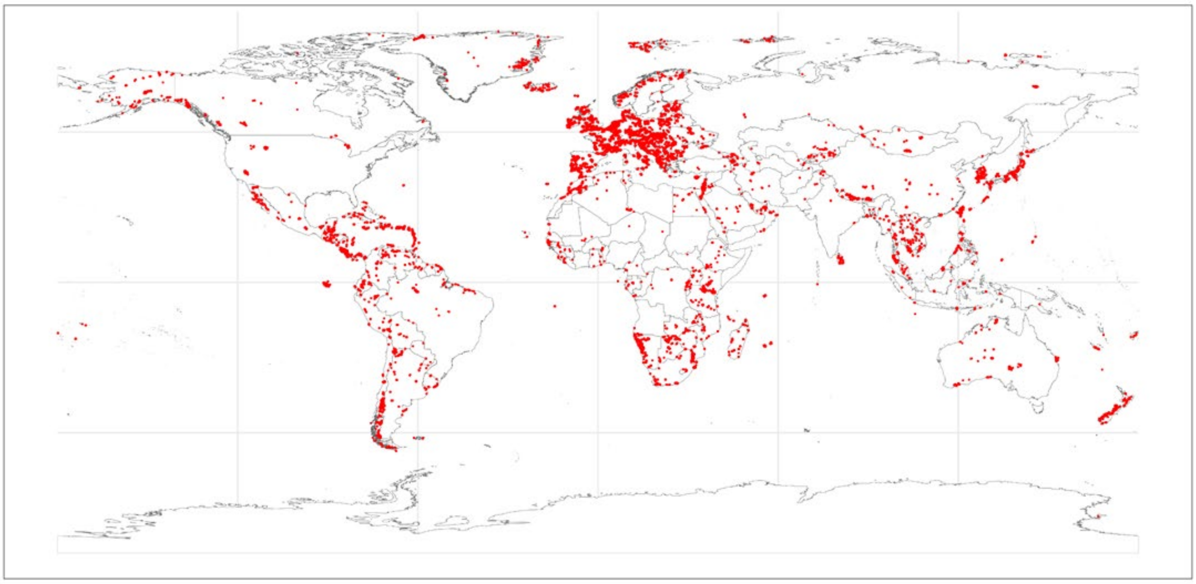

**Supplementary Figure S3. Global map of all photos (in red) downloaded from Flickr and included in the final sample.**

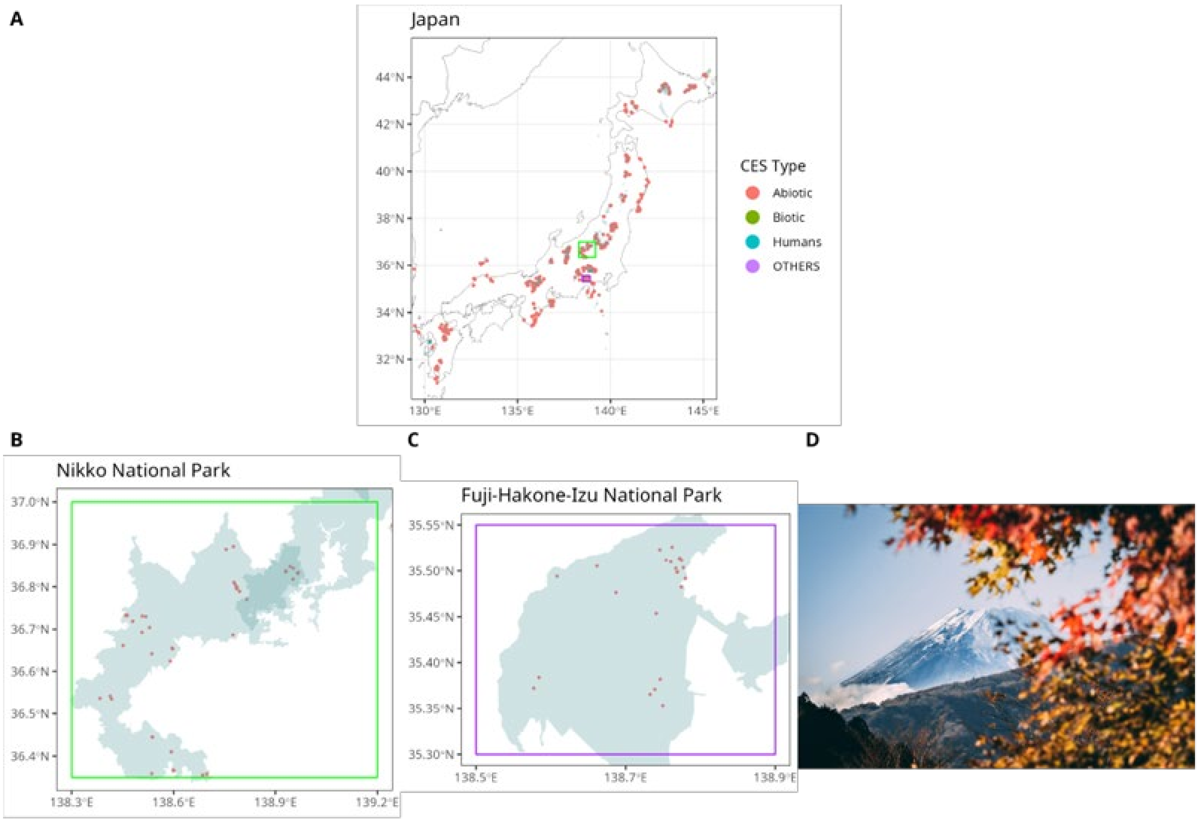


**Supplementary Figure S4. Maps of all photos sampled in Japan (A) and labelled by their CES type.** Spatial range of sampled PAs are added as a green overlay. Photos taken in popular PAs are highlighted, with Nikko National Park (B, in green), and Fuji-Hakone-Izu National Park (C, in purple) as examples. A sample photo (D) is also provided.

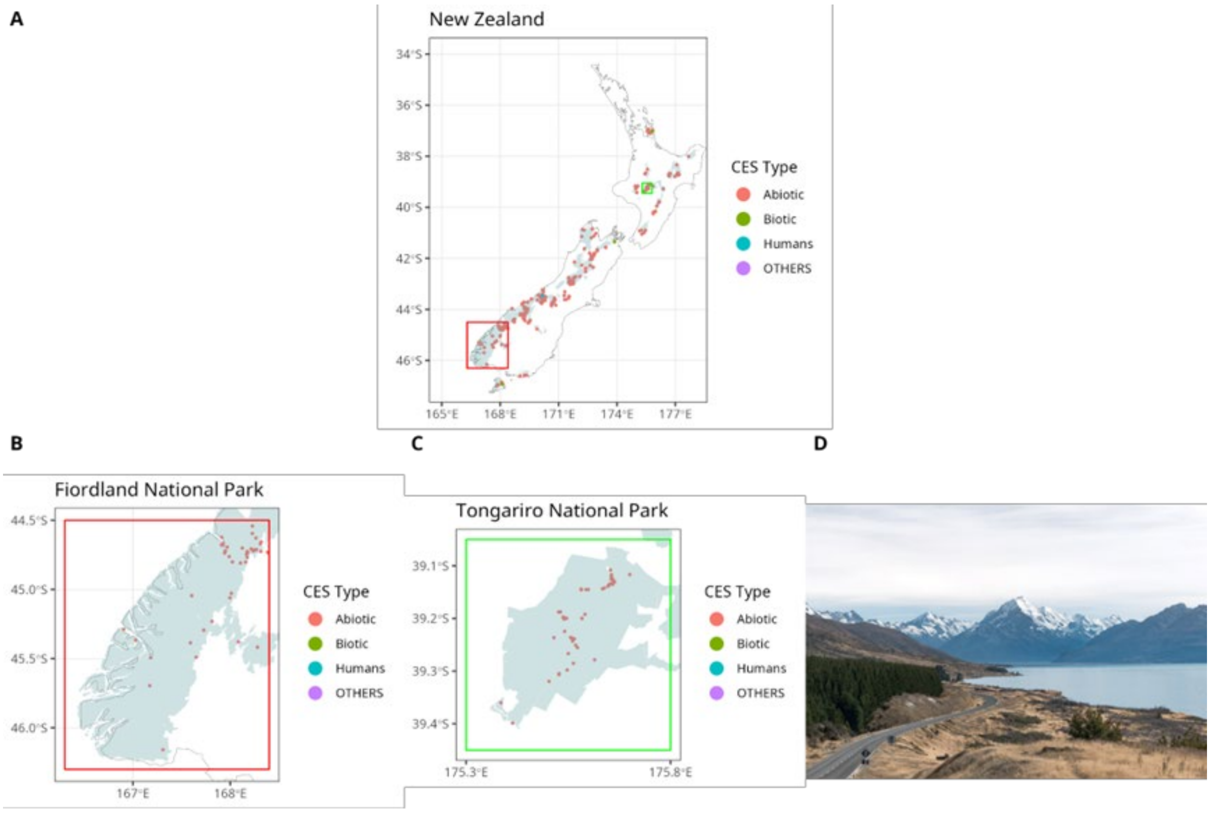


**Supplementary Figure S5. Maps of all photos sampled in New Zealand (A) and labelled by their CES type.** Spatial range of sampled PAs are added as a green overlay. Photos taken in popular PAs are highlighted, with Fiordland National Park (B, in red), and Tongariro National Park (C, in purple) as examples. A sample photo (D) is also provided.

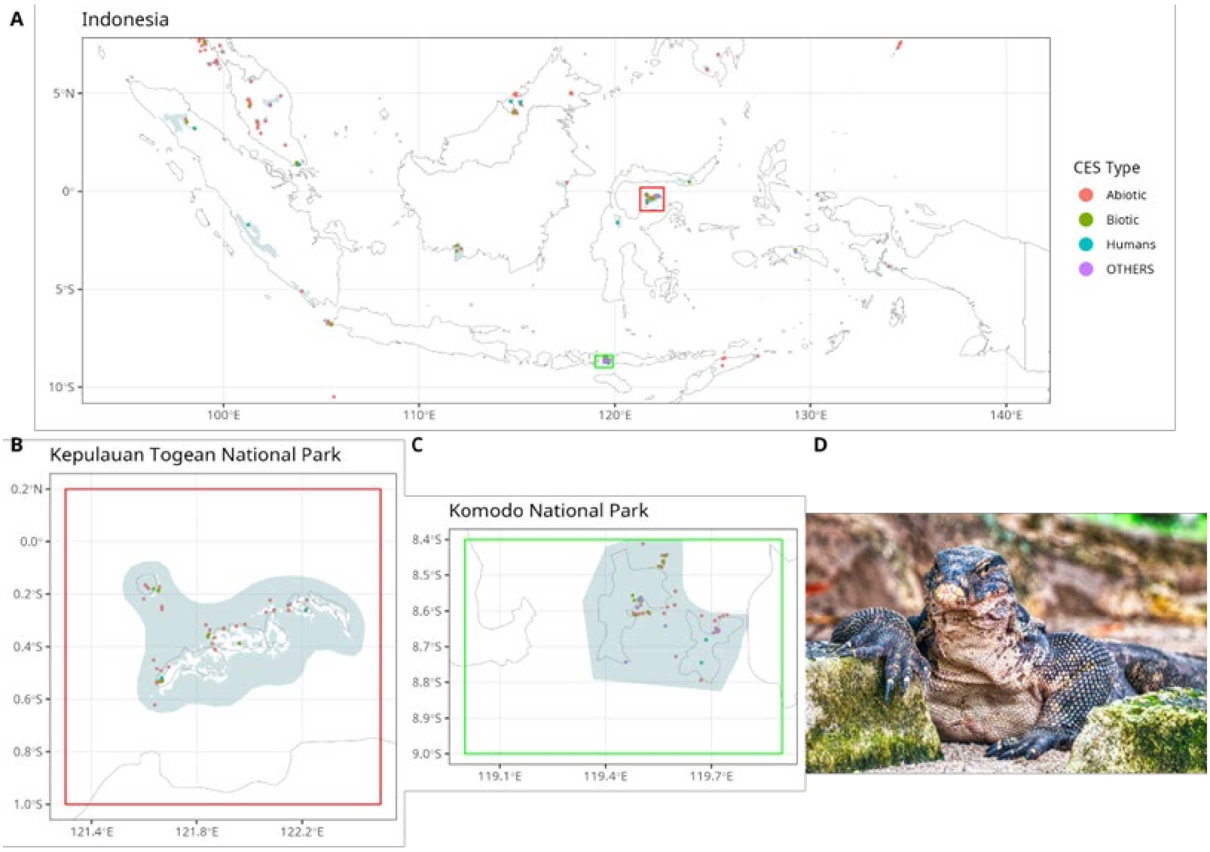


**Supplementary Figure S6. Maps of all photos sampled in Indonesia (A) and labelled by their CES type.** Spatial range of sampled PAs are added as a green overlay. Photos taken in popular PAs are highlighted, with Kepulauan Togean National Park (B, in red), and Komodo National Park (C, in green). A sample photo (D) is also provided.

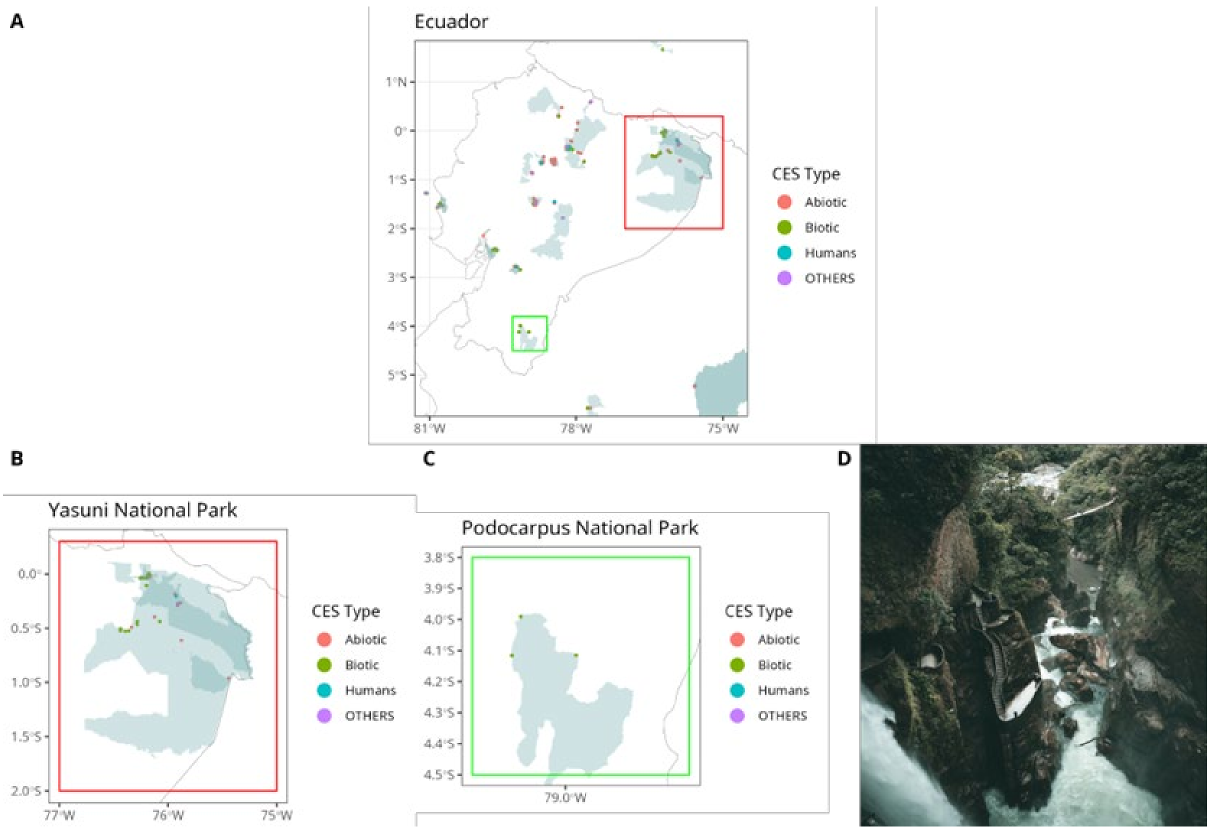


**Supplementary Figure S7. Maps of all photos sampled in Ecuador (A) and labelled by their CES type.** Spatial range of sampled PAs are added as a green overlay. Photos taken in popular PAs are highlighted, with Yasuní National Park (B, in red), and Podocarpus National Park (C, in green). A sample photo (D) is also provided.

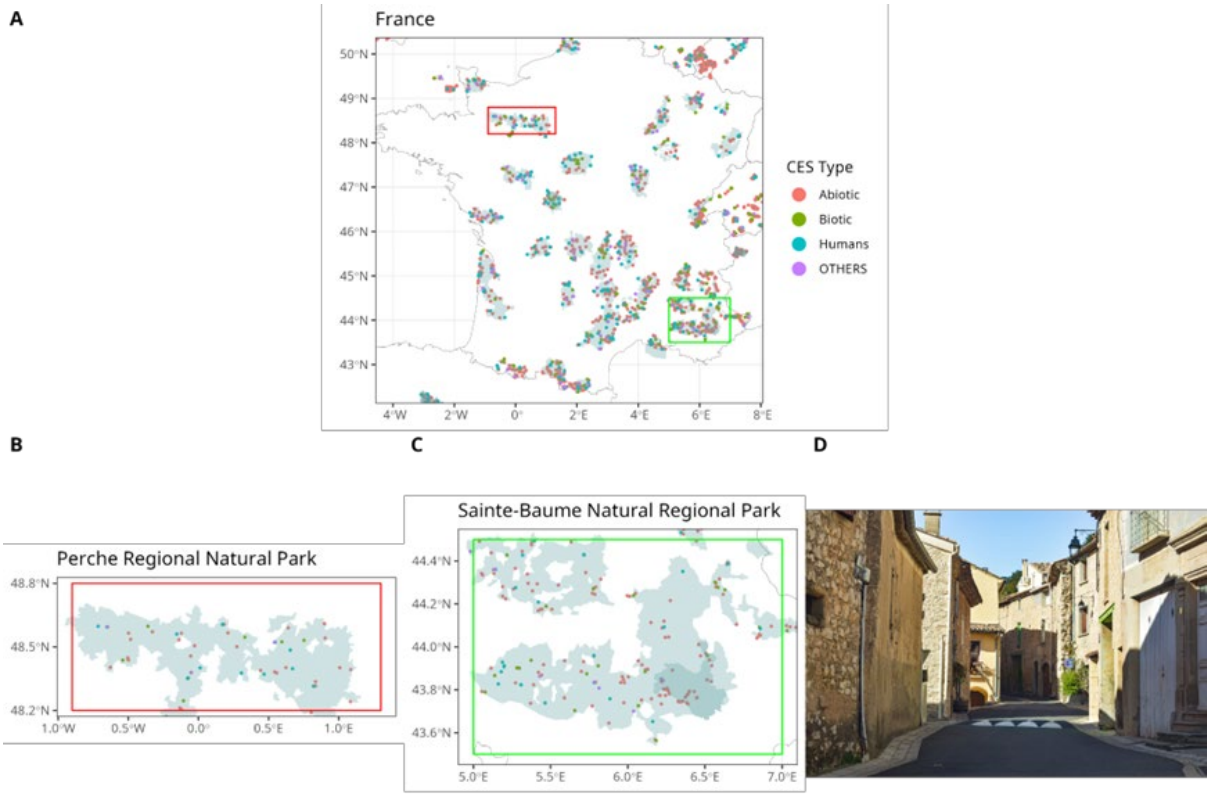


**Supplementary Figure S8. Maps of all photos sampled in France (A), and labelled by their CES type.** Spatial range of sampled PAs are added as a green overlay. Photos taken in popular PAs are highlighted, with Perche Regional Natural Park (B, in red), and Sainte-Baume Natural Regional Park (C, in green). A sample photo (D) is also provided.

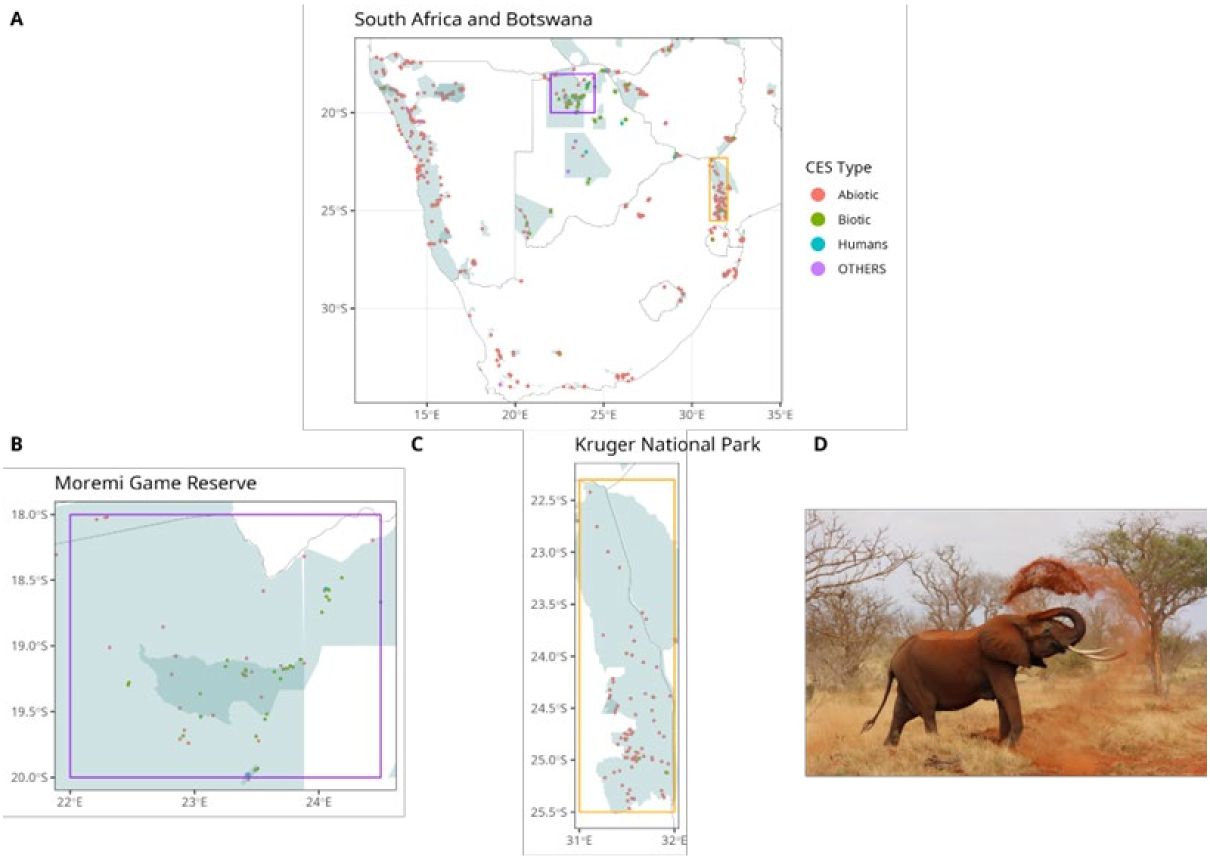


**Supplementary Figure S9. Maps of all photos sampled in South Africa and Botswana (A) and labelled by their CES type.** Spatial range of sampled PAs are added as a green overlay. Photos taken in popular PAs are highlighted, with Moremi Game Reserve (B, in red), and Kruger National Park (C, in green). A sample photo (D) is also provided.

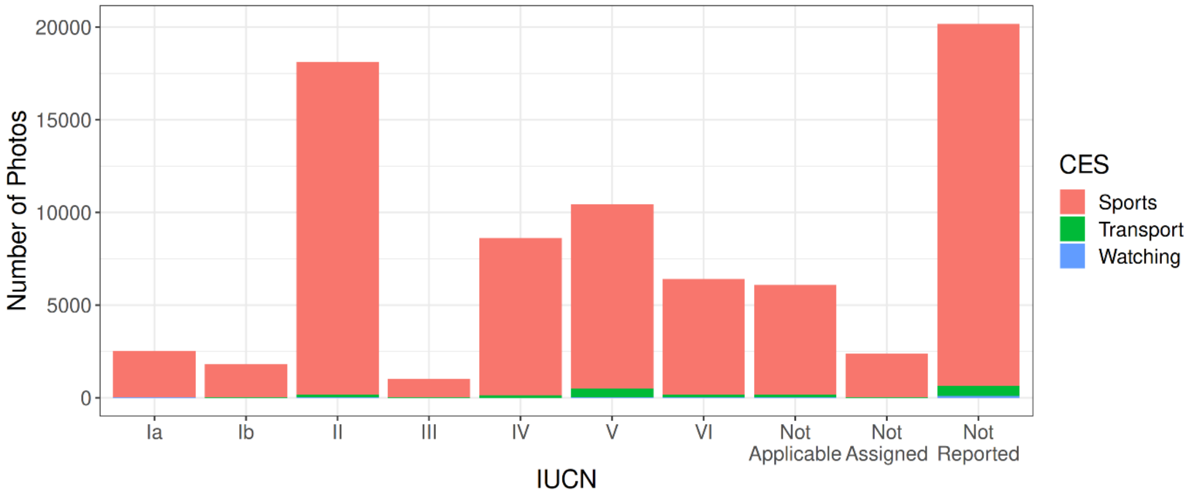


**Supplementary Figure S10. Number of photos in sampled PAs sorted by IUCN category, for Abiotic CES type.**

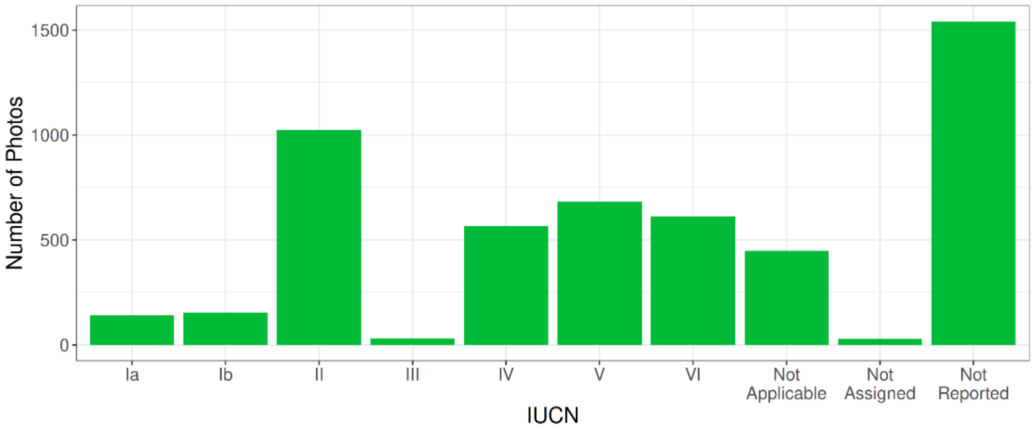


**Supplementary Figure S11. Number of photos in sampled PAs sorted by IUCN category, for Biotic CES type.**

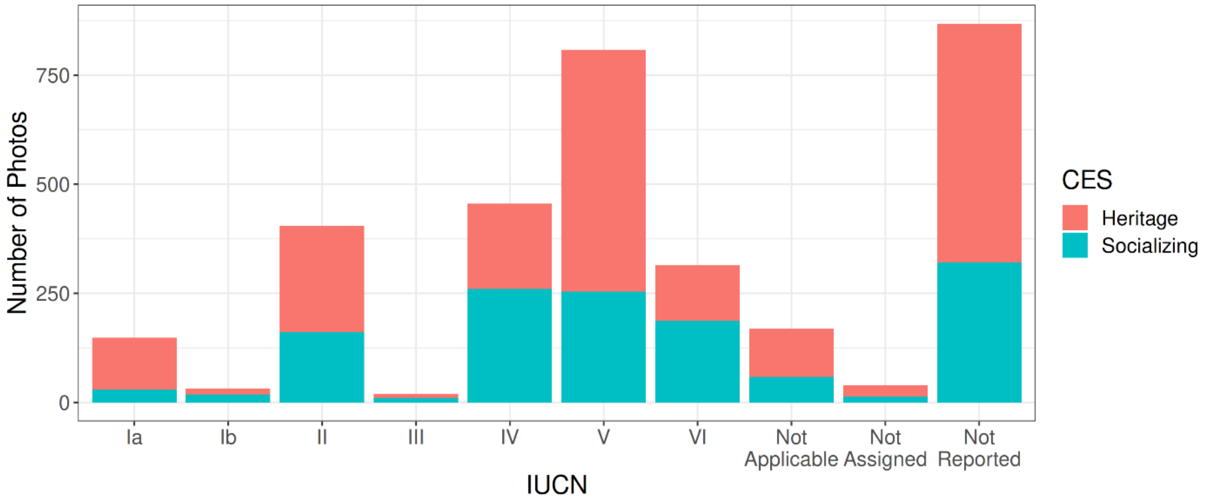


**Supplementary Figure S12. Number of photos in sampled PAs sorted by IUCN category, for Humans CES type.**

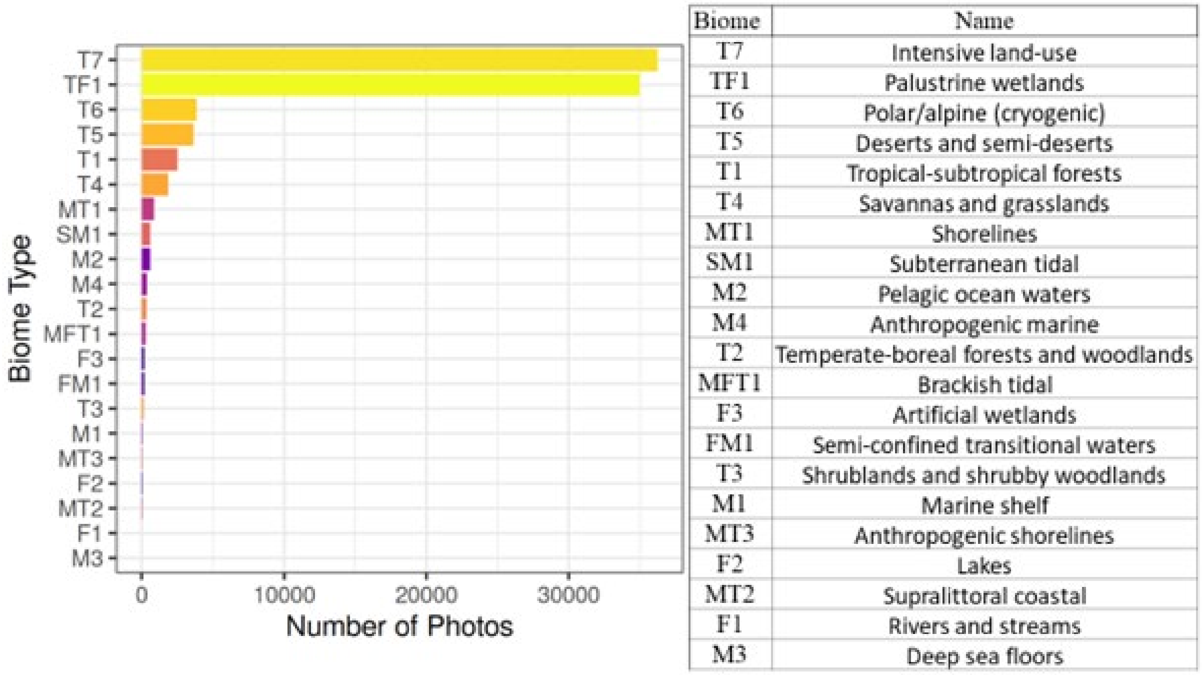


| Biome | Name |
|---|---|
| T7 | Intensive land-use |
| TF1 | Palustrine wetlands |
| T6 | Polar/alpine (cryogenic) |
| T5 | Deserts and semi-deserts |
| T1 | Tropical-subtropical forests |
| T4 | Savannas and grasslands |
| MT1 | Shorelines |
| SM1 | Subterranean tidal |
| M2 | Pelagic ocean waters |
| M4 | Anthropogenic marine |
| T2 | Temperate-boreal forests and woodlands |
| MFT1 | Brackish tidal |
| F3 | Artificial wetlands |
| FM1 | Semi-confined transitional waters |
| T3 | Shrublands and shrubby woodlands |
| M1 | Marine shelf |
| MT3 | Anthropogenic shorelines |
| F2 | Lakes |
| MT2 | Supralittoral coastal |
| F1 | Rivers and streams |
| M3 | Deep sea floors |

**Supplementary Figure S13. Number of photos taken in each biome type in the global sample (left), and the IUCN Global Ecosystem Typology name for each biome (right).**

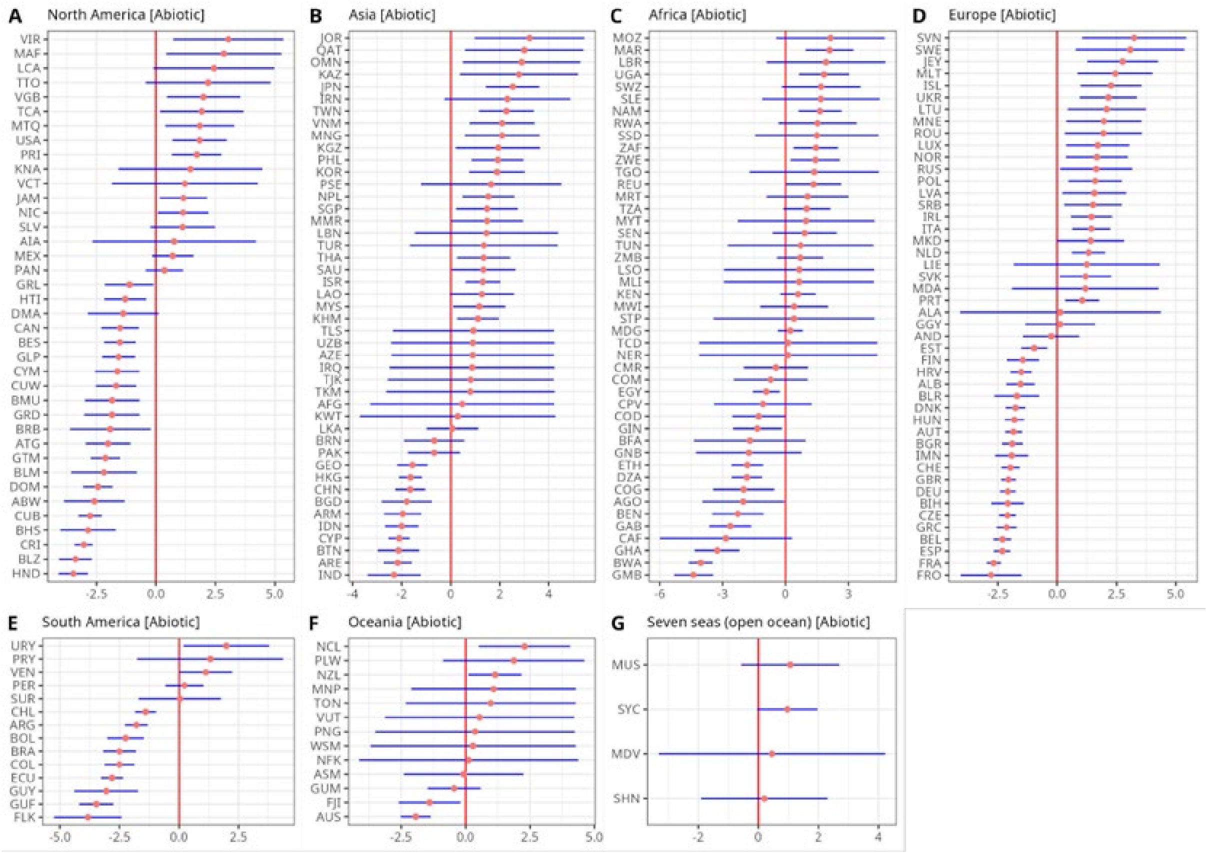


**Supplementary Figure S14. Random intercepts in the Abiotic mixed-effects model for all sampled countries worldwide, as grouped by continents and sorted by decreasing values in each continent.** Error bars (in blue) for all countries indicate standard errors of their estimates. Countries are referenced by their ISO 3166-1 alpha-3 codes.

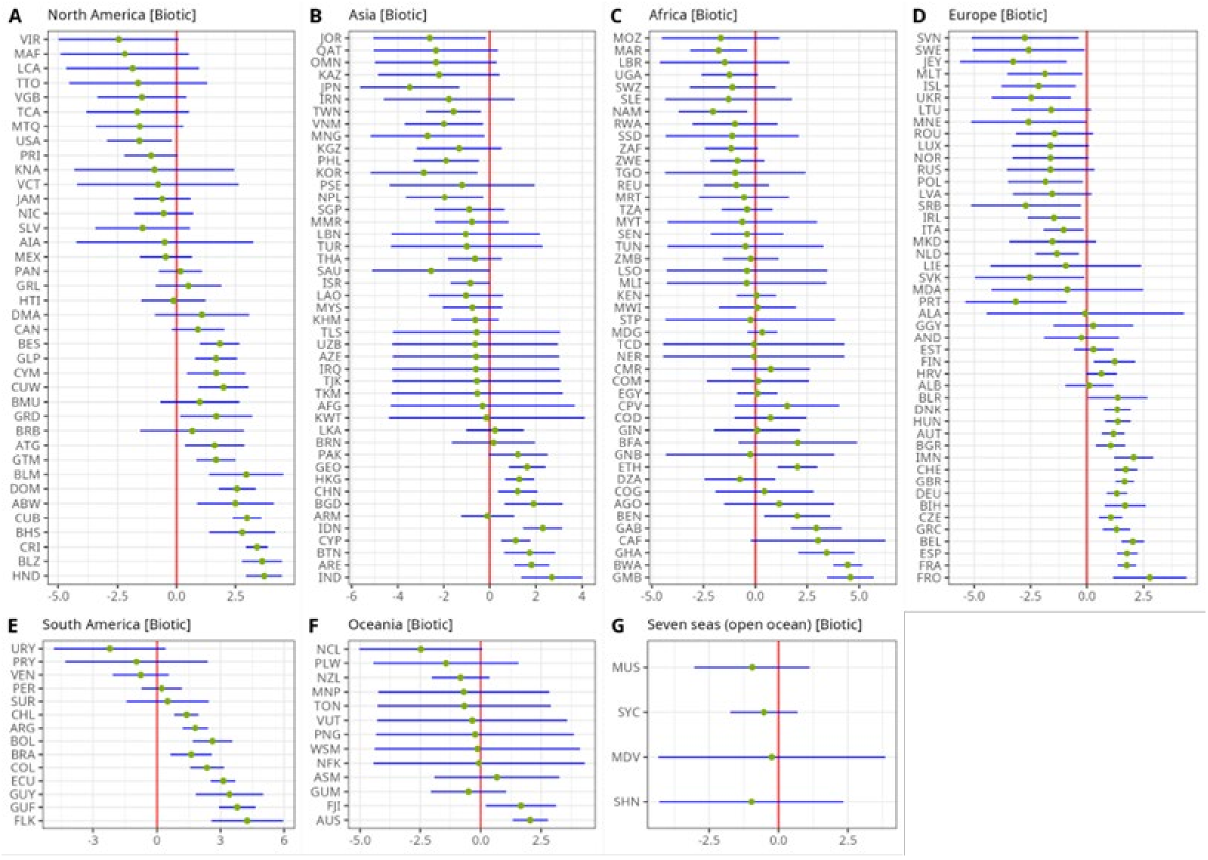


**Supplementary Figure S15. Random intercepts in the Biotic mixed-effects model for all sampled countries worldwide, as grouped by continents and sorted by decreasing values in the Abiotic model (S11 Fig) in each continent.** Error bars (in blue) for all countries indicate standard errors of their estimates. Countries are referenced by their ISO 3166-1 alpha-3 codes.

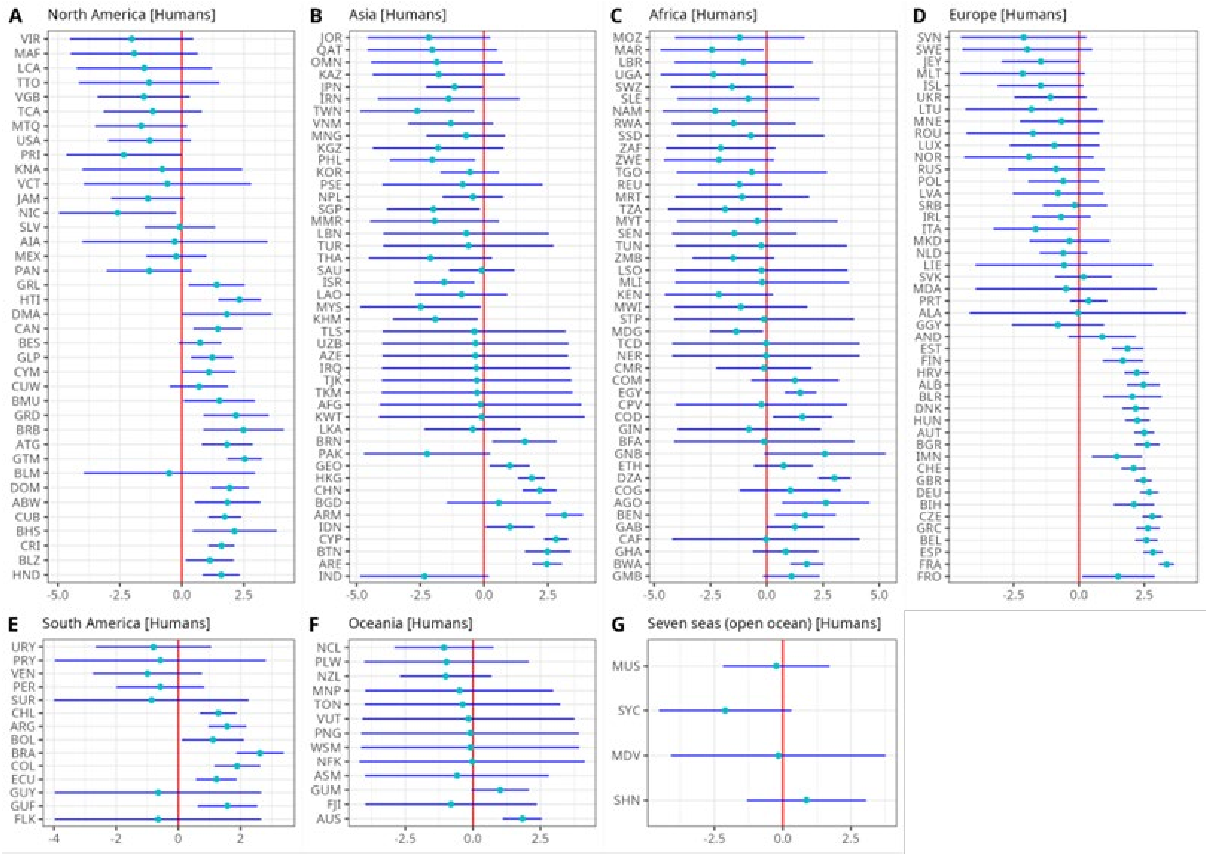


**Supplementary Figure S16. Random intercepts in the Humans mixed-effects model for all sampled countries worldwide, as grouped by continents and sorted by decreasing values in the Abiotic model (S11 Fig) in each continent.** Error bars (in blue) for all countries indicate standard errors of their estimates. Countries are referenced by their ISO 3166-1 alpha-3 codes.

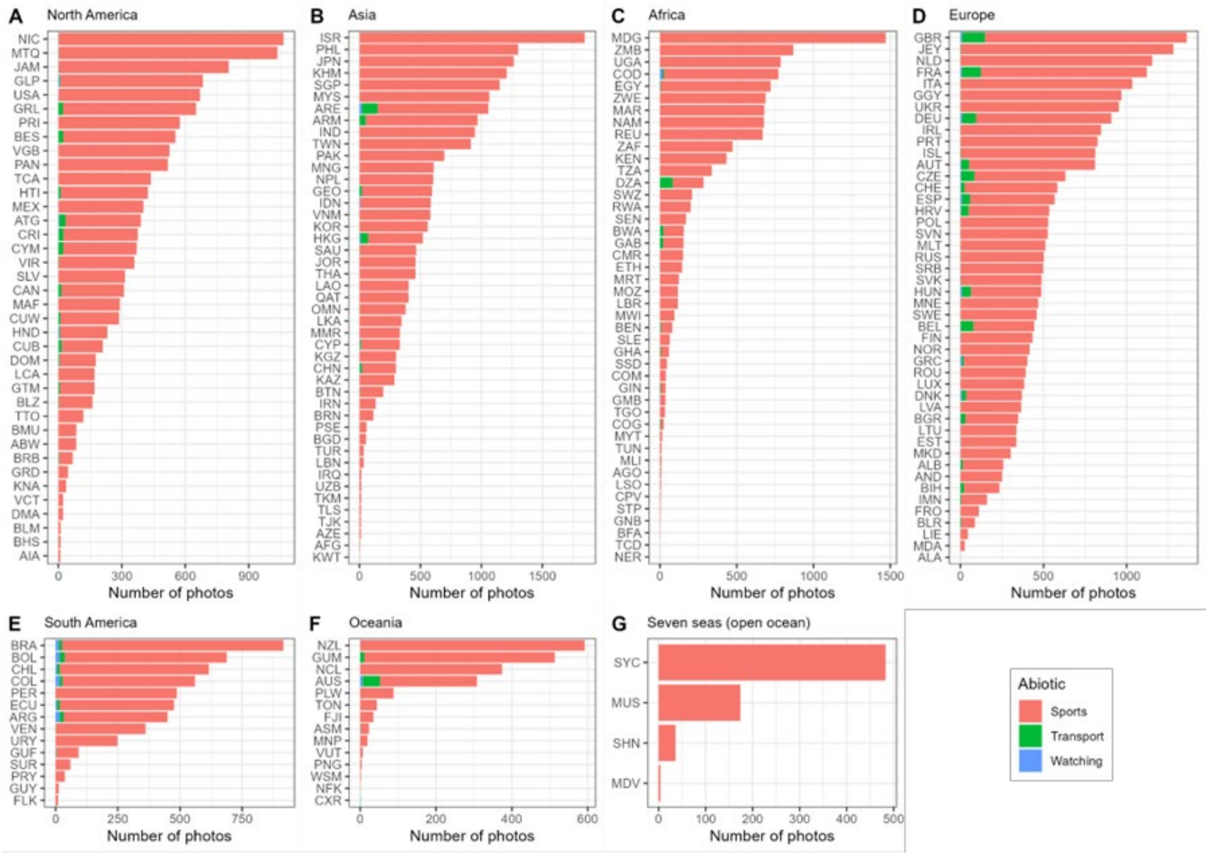


**Supplementary Figure S17. Number of photos of each subcategory of Abiotic CES type in each country separated by continent.**

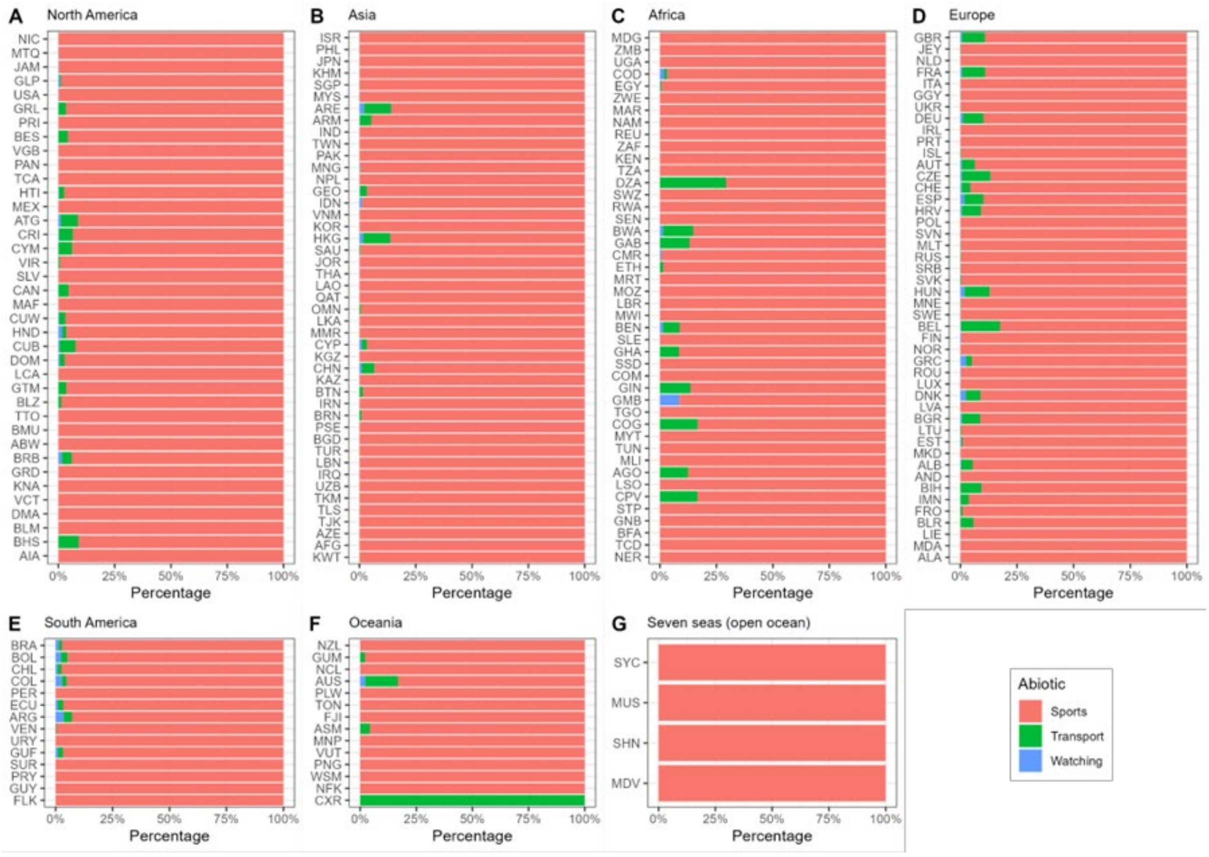


**Supplementary Figure S18. Percentage of photos of each subcategory of Abiotic CES type in each country separated by continent.**

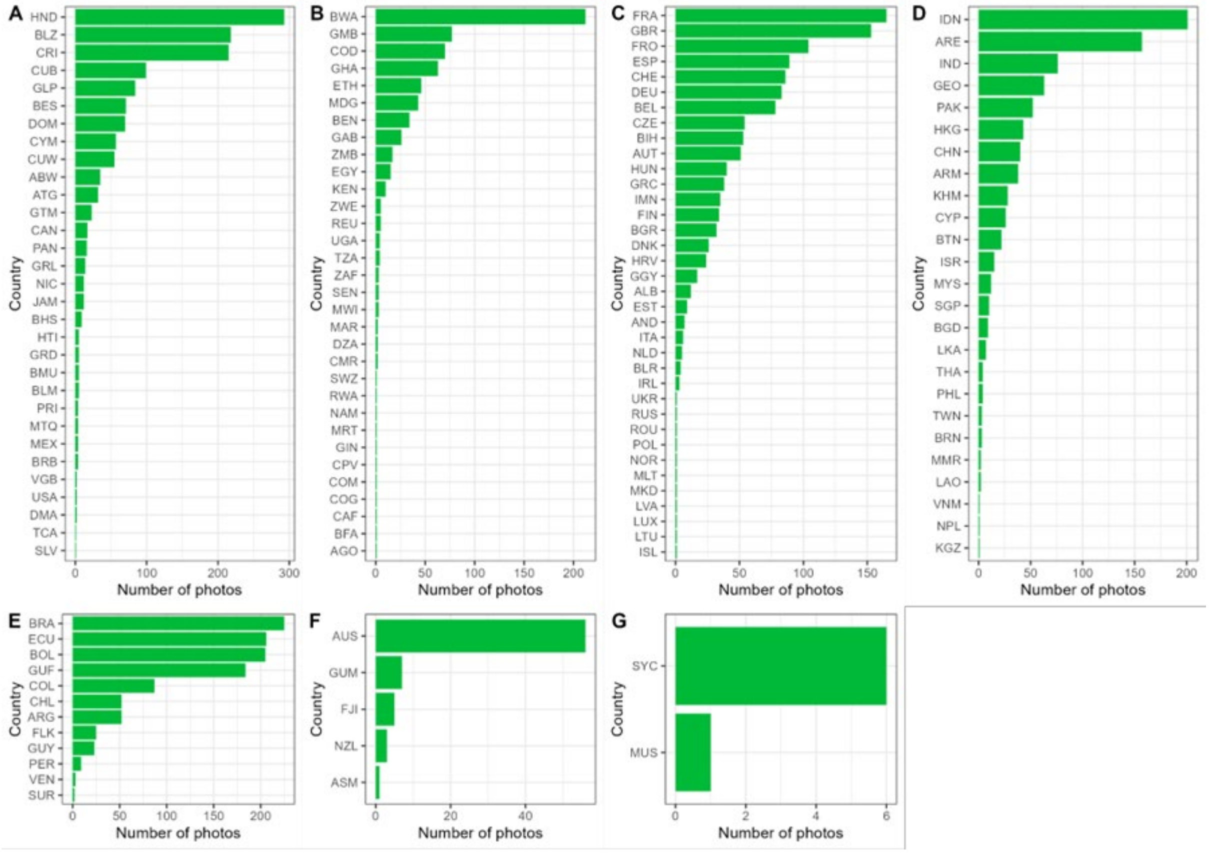


**Supplementary Figure S19. Number of photos of Biotic CES type in each country separated by continent.**

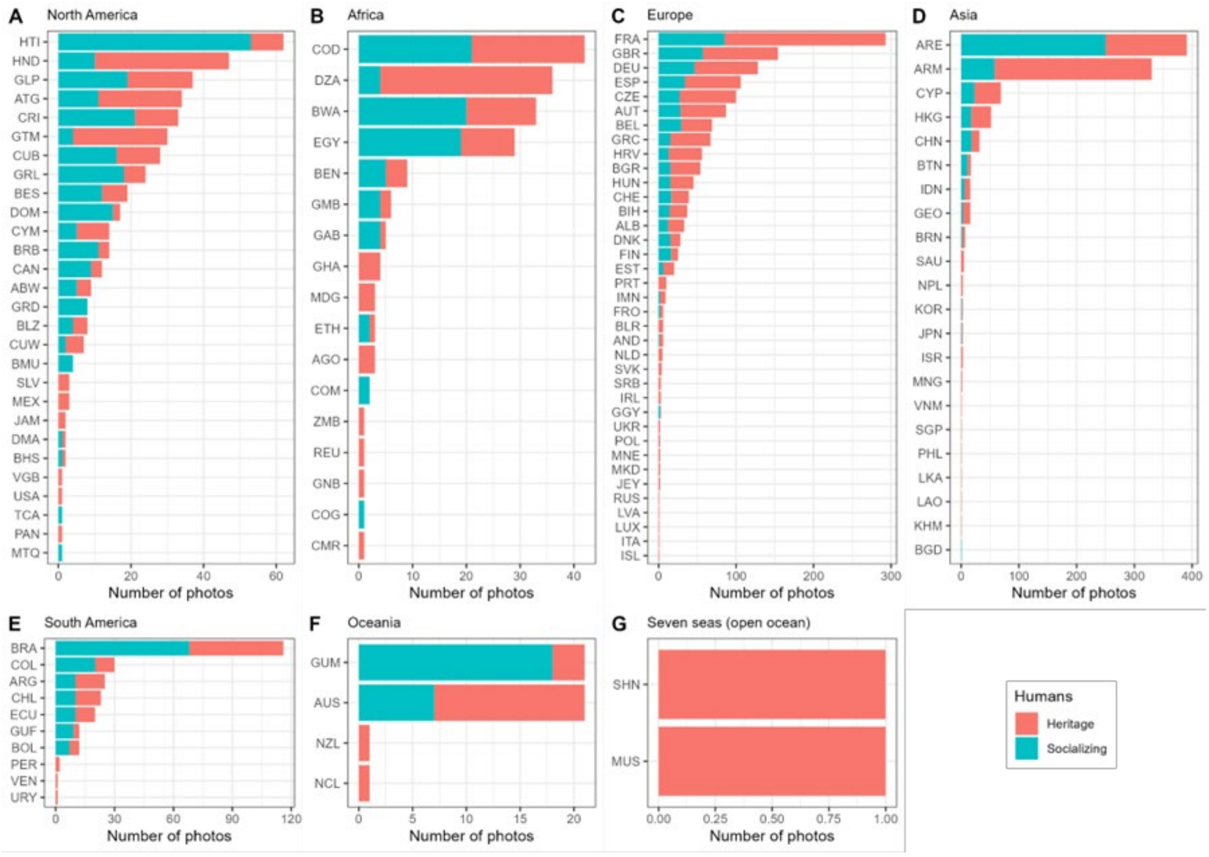


**Supplementary Figure S20. Number of photos of each subcategory of Humans CES type in each country separated by continent.**

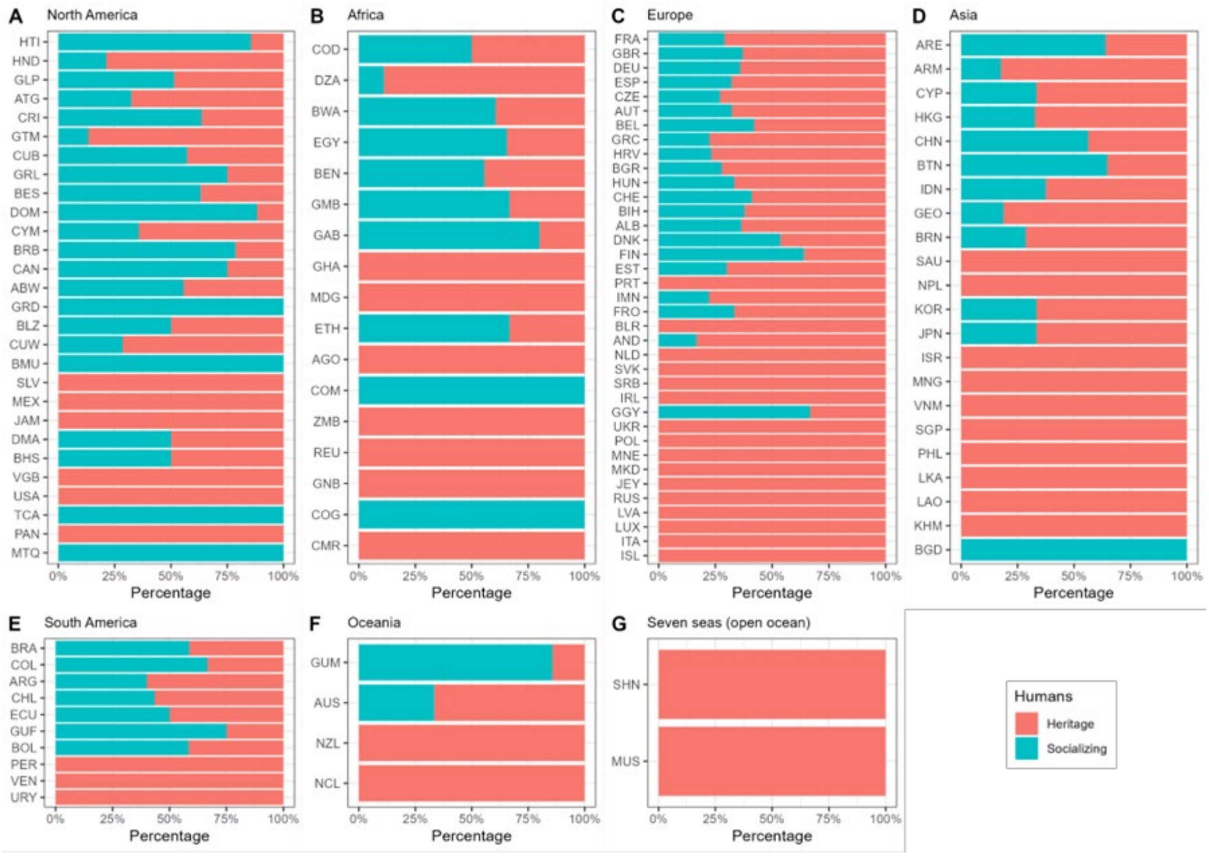


**Supplementary Figure S21. Percentage of photos of each subcategory of Humans CES type in each country separated by continent.**

**Supplementary Table S1. Summary of all three generalized linear regression models for biome type.**

| | abiotic | | | biotic | | | humans | | |
|---|---|---|---|---|---|---|---|---|---|
| *Predictors* | *Odds Ratios* | *CI* | *p* | *Odds Ratios* | *CI* | *p* | *Odds Ratios* | *CI* | *p* |
| (Intercept) | 34.09 | 24.09 – 48.23 | **<0.001** | 0.02 | 0.01 – 0.02 | **<0.001** | 0.01 | 0.00 – 0.01 | **<0.001** |
| T7-3: Plantations | 0.84 | 0.70 – 1.01 | 0.068 | 1.08 | 0.86 – 1.35 | 0.514 | 1.32 | 0.93 – 1.86 | 0.122 |
| T7-4: Urban and Industrial | 0.70 | 0.61 – 0.81 | **<0.001** | 1.05 | 0.88 – 1.26 | 0.563 | 1.92 | 1.54 – 2.38 | **<0.001** |
| T7-5: Semi-natural Old Fields | 0.74 | 0.65 – 0.85 | **<0.001** | 1.24 | 1.06 – 1.46 | **0.007** | 1.54 | 1.23 – 1.94 | **<0.001** |
| TF1-2: Temp Forested Wetlands | 0.89 | 0.73 – 1.07 | 0.219 | 1.15 | 0.89 – 1.50 | 0.287 | 1.21 | 0.93 – 1.56 | 0.151 |
| TF1-6: Boreal Temperate Bogs | 1.43 | 1.09 – 1.87 | **0.010** | 0.74 | 0.52 – 1.06 | 0.101 | 0.80 | 0.55 – 1.17 | 0.258 |
| TF1-7: Boreal Temperate Ferns | 0.93 | 0.74 – 1.18 | 0.555 | 1.06 | 0.76 – 1.48 | 0.737 | 1.17 | 0.86 – 1.59 | 0.307 |
| **Random Effects** | | | | | | | | | |
| $\sigma^2$ | 3.29 | | | 3.29 | | | 3.29 | | |
| $\tau_{00}$ | 0.78 $_{\text{ProtectedPlace:Country}}$ | | | 1.31 $_{\text{ProtectedPlace:Country}}$ | | | 0.68 $_{\text{ProtectedPlace:Country}}$ | | |
| | 5.04 $_{\text{Country}}$ | | | 5.10 $_{\text{Country}}$ | | | 4.44 $_{\text{Country}}$ | | |
| ICC | 0.64 | | | 0.66 | | | 0.61 | | |
| N | 2795 $_{\text{ProtectedPlace}}$ | | | 2795 $_{\text{ProtectedPlace}}$ | | | 2795 $_{\text{ProtectedPlace}}$ | | |
| | 205 $_{\text{Country}}$ | | | 205 $_{\text{Country}}$ | | | 205 $_{\text{Country}}$ | | |
| Observations | 87016 | | | 87016 | | | 87016 | | |
| Marginal $R^2$ / Conditional $R^2$ | 0.003 / 0.640 | | | 0.001 / 0.661 | | | 0.007 / 0.611 | | |

**Supplementary Table S2. Abiotic CES category and subcategories with definitions.**

| CES Category | Proposed definition in this paper | Definition in CICES (Haines-Young & Potschin, 2018) |
|---|---|---|
| Abiotic | Photos containing:<br>1. nonliving parts of an ecosystem, or<br>2. human activities that interact with the nonliving parts of an ecosystem | **Cultural (Abiotic)**<br>"Direct, in-situ and outdoor interactions with natural physical systems that depend on presence in the environmental setting" |
| Transport | Photos involve human activities that directly interact with the abiotic environment through some mode of transport. These can include motor vehicles, or other forms of transportation such as airplanes and trains. | **Cultural (Abiotic) — Recreation**<br>"things in the physical environment that we can study or think about"<br>"natural, abiotic characteristics of nature that … enable intellectual activities" |
| Sports | Photos depicting humans that engage in some recreational activity that can only be done in nature. These include sports such as skiing and hiking. | **Cultural (Biotic) — Recreation**<br>"using the environment for sport and recreation; using nature to help stay fit"<br>"the biophysical characteristics or qualities of species or ecosystems (settings/ cultural spaces) … that are engaged with, used or enjoyed in ways that require physical and cognitive effort" |
| Watching | Photos emphasizing human interaction with the abiotic environment through means that do not involve direct physical contact. This includes observing the natural environment for various reasons, such as aesthetic appreciation, or spiritual/religious reasons. | **Cultural (Abiotic) — Ecotourism**<br>"things in the physical environment that we can study or think about"<br>"natural, abiotic characteristics of nature that … enable active or passive physical and experiential interactions" |

**Supplementary Table S3. Biotic CES category and subcategory with definitions.**

| CES Category | Proposed definition in this paper | Definition in CICES (Haines-Young & Potschin, 2018) |
|---|---|---|
| Biotic | Photos containing:<br>1. tags that mainly describe a specific animal or plant, or<br>2. humans interacting with a specific animal or plant as the focus | **Cultural (Biotic)**<br>"Direct, in-situ and outdoor interactions with living systems that depend on presence in the environmental setting" |
| Watching | Same as Biotic | **Cultural (Biotic) — Ecotourism**<br>“watching plants and animals where they live; using nature to destress"”<br>“the biophysical characteristics or qualities of species or ecosystems (settings/cultural spaces)…. that are viewed/ observed by people or enjoyed in other passive ways by virtue of sounds and smells etc.” |

**Supplementary Table S4. Humans CES category and subcategories with definitions.**

| CES Category | Proposed definitions in this paper | Definition in Chan et al. (2016) |
|---|---|---|
| Humans | Photos containing:<br>1. human subjects as the key focus, or<br>2. activities involving interactions between humans, or<br>3. man-made locations | “Relational values (involving the human collective) … pertain to all manner of relationships between people and nature, including relationships that are between people but involve nature” |
| Heritage | Photos mainly highlight old buildings of historical importance. | “Being in nature provides a vehicle for me to connect with people (Social cohesion)" |
| Socialising | Photos mainly highlight interactions between people. | “Place is important to my people, to who we are as a people (Cultural identity)" |

**Supplementary Table S5. Number of photos of each biome type.**

| | Biome Type | Number of Photos |
|---|---|---|
| 1 | T7-5: Semi-natural old fields | 19445 |
| 2 | TF1-2: Temp forested wetlands | 15346 |
| 3 | T7-4: Urban and industrial | 10565 |
| 4 | TF1-7: Boreal temperate fens | 8282 |
| 5 | T7-3: Plantations | 4165 |
| 6 | TF1-6: Boreal temperate bogs | 4050 |
| 7 | TF1-4: Seasonal floodplain marshes | 3462 |
| 8 | TF1-1: Trop flooded peat forests | 2667 |
| 9 | T6-4: Temp alpine grasslands | 2435 |
| 10 | T5-5: Hyper-arid deserts | 2260 |

| | | |
|---|---|---|
| 11 | T4-2: Pyric tussock savannas | 1768 |
| 12 | T1-3: Trop montane rainforests | 1565 |
| 13 | T7-2: Sown pastures and fields | 1552 |
| 14 | TF1-3: Permanent marshes | 989 |
| 15 | MT1-2: Muddy shores | 834 |
| 16 | T5-1: Semi-desert steppe | 742 |
| 17 | T6-3: Polar tundra | 681 |
| 18 | T1-1: Trop lowland rainforests | 649 |
| 19 | SM1-3: Sea caves | 598 |
| 20 | M2-1: Epipelagic waters | 561 |
| 21 | T7-1: Croplands | 537 |
| 22 | T6-2: Polar alpine rock | 433 |

| | | |
|---|---|---|
| 23 | T6-5: Trop alpine grassland | 299 |
| 24 | T5-2: Succulent Thorny deserts | 297 |
| 25 | MFT1-2: Intertidal forests | 261 |
| 26 | T5-4: Cool temperate deserts | 253 |
| 27 | M4-2: Marine aquafarms | 248 |
| 28 | T2-4: Warm temp rainforests | 226 |
| 29 | T1-2: Trop dry forests | 213 |
| 30 | TF1-5: Episodic arid floodplains | 186 |
| 31 | M4-1: Submerged artificial structures | 144 |
| 32 | FM1-2: Riverine estuaries and bays | 132 |
| 33 | T3-4: Rocky pavements | 123 |
| 34 | T2-1: Boreal montane forests | 109 |

| | | |
|---|---|---|
| 35 | T1-4: Trop heath forests | 78 |
| 36 | MT3-1: Artificial shores | 76 |
| 37 | F3-5: Canals and drains | 74 |
| 38 | M1-8: Subtidal mud plains | 73 |
| 39 | F3-2: Constructed lacustrine wetlands | 71 |
| 40 | T5-3: Sclerophyll hot deserts | 67 |
| 41 | F3-3: Rice paddies | 45 |
| 42 | FM1-3: Closed open inlets | 42 |
| 43 | MT2-1: Coastal shrublands and grasslands | 41 |
| 44 | F2-6: Perm salt lakes | 37 |
| 45 | MFT1-3: Coastal saltmarsh | 34 |
| 46 | F1-6: Episodic arid rivers | 33 |

| | | |
|---|---|---|
| 47 | T4-4: Temp woodlands | 29 |
| 48 | T4-3: Hummock savannas | 28 |
| 49 | M3-1: Continental slopes | 25 |
| 50 | M2-5: Sea ice | 24 |
| 51 | T3-1: Seas dry trop shrublands | 23 |
| 52 | M2-2: Mesopelagic ocean waters | 21 |
| 53 | T4-5: Temperate grasslands | 21 |
| 54 | F2-9: Geothermal wetlands | 19 |
| 55 | FM1-1: Deepwater coastal inlets | 16 |
| 56 | SM1-2: Anchialine pools | 13 |
| 57 | F2-4: Freeze-thaw lakes | 10 |
| 58 | M1-2: Kelp forests | 6 |

| | | |
|---|---|---|
| 59 | F2-8: Artesian springs oases | 5 |
| 60 | F3-4: Freshwater aquafarms | 5 |
| 61 | T4-1: Trophic savannas | 5 |
| 62 | T6-1: Permanent snow | 5 |
| 63 | F2-1: Large perm lakes | 4 |
| 64 | T3-2: Seas dry temp shrublands | 4 |
| 65 | M3-5: Deepwater biogenic beds | 1 |
| 66 | M3-6: Hadal | 1 |
| 67 | M3-7: Chemosynthetic | 1 |
| 68 | MFT1-1: River deltas | 1 |
| 69 | MT1-3: Sandyshores | 1 |
